# Use of Psychotropic Medications and Illegal Drugs, and Related Consequences Among French Pharmacy Students – SCEP Study: A Nationwide Cross-Sectional Study

**DOI:** 10.3389/fphar.2018.00725

**Published:** 2018-07-17

**Authors:** David Balayssac, Bruno Pereira, Maxime Darfeuille, Pierre Cuq, Laurent Vernhet, Aurore Collin, Brigitte Vennat, Nicolas Authier

**Affiliations:** ^1^Inserm U1107, NEURO-DOL, Faculté de Pharmacie, Laboratoire de Toxicologie, Université Clermont Auvergne, CHU Clermont-Ferrand, Délégation à la Recherche Clinique et à l’Innovation, Clermont-Ferrand, France; ^2^CHU Clermont-Ferrand, Délégation à la Recherche Clinique et à l’Innovation, Clermont-Ferrand, France; ^3^Faculté de Pharmacie, Laboratoire de Toxicologie, Université Clermont Auvergne, Clermont-Ferrand, France; ^4^Institut des Biomolécules Max Mousseron (IBMM), UMR 5247, CNRS, ENSCM, Faculté de Pharmacie, Université de Montpellier, Montpellier, France; ^5^UMR Inserm 1085, Institut de Recherche sur la Santé, l’Environnement et le Travail (IRSET), Université de Rennes 1, Rennes, France; ^6^Inserm U1107, NEURO-DOL, Faculté de Pharmacie, Laboratoire de Toxicologie, Université Clermont Auvergne, Clermont-Ferrand, France; ^7^ACCePPT, Faculté de Pharmacie, Université Clermont Auvergne, Clermont-Ferrand, France; ^8^Inserm U1107, NEURO-DOL, Université Clermont Auvergne, Faculté de Médecine, CHU Clermont-Ferrand, Pharmacologie Médicale, Clermont-Ferrand, France

**Keywords:** psychotropic drugs, street drugs, pharmacy student, self-medication, comorbidity

## Abstract

**Background:** The use of psychotropic medications and illegal drugs is a worldwide public health issue, leading to addiction, psychiatric and somatic disorders, and death. Pharmacy students are more exposed to psychotropic medications than other students (non-medical), which could lead to an overuse. The main objective of this study was to assess the prevalence of psychotropic drug use (medications and illegal drugs) by French pharmacy students, by carrying out a nationwide cross-sectional study. The relation of these medications and illegal drug use with several comorbidities and academic achievement was also assessed.

**Methods:** This online survey was performed by emails sent to all French pharmacy faculties over a period of 66 days (March 16, 2016 to May 20, 2016). The survey assessed the prevalence of uses of psychotropic medications and illegal drugs during the last 3 months. These uses were compared to student characteristics (personal and university) and comorbidities (anxiety, depression, stress, and fatigue).

**Results:** Of the 2,609 questionnaires received, 2,575 were completed and useable for the analysis. Among French pharmacy students and during the 3 last months, 9.4% have used psychotropic medications, 21.5% illegal drugs and 3.3% both psychotropic medications and illegal drugs. Psychotropic medications were used in the cases of a medical prescription (49.0%), a self-medication (42.4%) or a non-medical intent (26.3%). Stress scores of the last 7 days were higher for psychotropic medication users compared to non-users and illegal drug users. Proportions of anxiety and depression at the time of answer were higher for psychotropic medication users than for non-users and illegal drug users. Fatigue scores of the last 7 days were lower for illegal drug users compared to non-users and self-medicated students. Annual average marks of the last year, attendance and perception of study difficulty were lower for illegal drug users than for non-users.

**Conclusion:** French pharmacy students were less exposed to psychotropic medications and illegal drugs than the general French population. However, in comparison to other students in other countries, the use of psychotropic medications seemed to be lower, but with a proportionally higher use of anxiety/sedative medications and a lower use of opioid medications.

## Introduction

The use of psychotropic medications and illegal drugs is a worldwide public health issue leading to addiction, psychiatric and somatic disorders, and death (United Nations Office on Drugs and Crime [UNODC], 2016). Among psychotropic medications, benzodiazepines, Z-drugs (zolpidem and zopiclone) and opioids are the main drugs used and misused (Casati et al., 2012). The prevalence of use of medically prescribed benzodiazepines was about 9.7% of young adults in 2013 in France (Bénard-Laribière et al., 2016). Benzodiazepines and Z-drugs are used by more than 5.2% of American people between the ages of 18 and 80 years old (Soyka, 2017). Besides the risk of addiction for chronic users, benzodiazepines are responsible for several adverse effects such as drowsiness, lethargy, fatigue, excessive sedation, stupor, “hangover effects" the next day, disturbances of concentration and attention, symptom rebound after discontinuation (i.e., recurrence of the original disorder, most commonly a sleep disorder), hypotonia, ataxia, impaired driving ability, falls (fractures) and death from overdose (3.1 deaths per 100,000 American adults in 2013) (Bachhuber et al., 2016; Soyka, 2017). Although initially presented as superior to benzodiazepines, Z-drugs are not likely different to benzodiazepines regarding either effectiveness or safety (Holbrook, 2004). Misuse of opioid medications is currently a growing problem worldwide (Morley et al., 2017). In the United States in 2005, 4–5% of the adult population (9.6–11.5 million people) were prescribed long-term opioid therapy (Boudreau et al., 2009). Thanks to their pain relieving and sedative properties opioid medications may also produce feelings of pleasure, contentedness, and satisfaction, which may lead to misuse, abuse and addiction (Pergolizzi et al., 2017). Young individuals are more at risk of inappropriate opioid use (Peck et al., 2016). Misuse and addiction may lead to death by overdose. In the United States, the epidemic of opioid-overdose led to 42,000 deaths in 2016 (Oquendo and Volkow, 2018).

In 2014, it was estimated that 5% of the world population between 15 and 64 years old have used at least one illegal drug (United Nations Office on Drugs and Crime [UNODC], 2016). Cannabis remains by far the most widely cultivated, trafficked and abused “illegal” (depending on the country) drug worldwide (WHO, 2018). But despite its wide availability, cannabis use is not safe. Cannabis addiction will affect 10% of individuals who start using cannabis and 25–50% of individuals who use cannabis daily (Sachs et al., 2015). Cannabis use can induce acute and long-term disorders, such as cardiovascular disorders (tachycardia, myocardial infarction and cardiomyopathies, etc.), respiratory disorders (airway inflammation, chronic bronchitis, emphysema, etc.), lung cancer, immunosuppression, sleep disorders (somnolence and decrease of the sleep quality, etc.), cognitive disorders (memory impairment, deficits in visual attention, verbal fluency, etc.) and substance use disorders (Sachs et al., 2015; Blanco et al., 2016). Cannabis use may be associated with psychiatric disorders, but data from the scientific literature remains more ambiguous (Sachs et al., 2015; Blanco et al., 2016; Hill, 2017).

Pharmacy studies in France last 6 years and begin with a difficult competitive exam at the end of the 1st year with a limited number of places for each university (*numerus clausus*). French pharmacy studies are aimed at preparing future pharmacists for several roles, such as prevention, screening, diagnosis, treatment and patient follow-up; the dispensing and administration of medications, products and medical devices, and giving pharmaceutical advice; guidance regarding the healthcare system and the medico-social sector; and health education (Journal République Française, 2009). French pharmacy students attend university courses on pharmacology, toxicology and clinical pharmacy, including psychotropic medications and illegal drugs. These courses integrate the benefit/risk ratio of psychotropic medication use, so that these future pharmacists will be able to protect their patients from drug misuse and abuse. The risk of illegal drug use and therapeutic strategies to prevent drug addiction are also taught to pharmacy students.

We hypothesize that this knowledge could have two consequences on student behavior. On the one hand, it is expected that these students would be better informed on risks and thus be protected against the use and misuse of these psychotropic medications and drugs. On the other hand, this knowledge may comfort students in the use of these drugs, as they consider that they know what they are doing and are aware of the risks. It is known that medical students tend to have greater knowledge of appropriate self-medication, are more confident and have greater awareness of the effects of self-medication, and tend to practice it more often and appropriately (Durrieu et al., 2007; James et al., 2008). Medical and pharmacy students have a high prevalence of self-medication practices (78–88%) (Lukovic et al., 2014; Patil et al., 2014; Alkhatatbeh et al., 2016). In India, the prevalence of self-medication amongst medical students increases from the first (50%) to the final year of study (90.5%) (Kasulkar and Gupta, 2015). In comparison, self-medication is about 35% in the adult Brazilian population (within the last 15 days) (Domingues et al., 2015), 20% in the adult Spanish populations (within the last 15 days) (Carrasco-Garrido et al., 2010), 50% in adolescents (meta-analysis) (Gualano et al., 2015) and 38% in the elderly (systematic review) (Jerez-Roig et al., 2014). Moreover, French pharmacy students have easy access to medications during their internships in community pharmacies and hospitals (pharmacy and clinical departments), and student jobs in community pharmacies. However, little is known of the prevalence of psychotropic medications and illegal drugs used by pharmacy students.

Thus, the main objective of this study was to assess the prevalence of psychotropic drug use (medications and illegal drugs) during the last 3 months and its related medical indication among French pharmacy students, by performing a nationwide cross-sectional study. The secondary objectives focused on the association between these medications (medical prescription and self-medication) and illegal drug use with several comorbidities (anxiety and depression at the time of the answer, stress and fatigue during the last 7 days) and academic achievement.

## Materials and Methods

### Study Design

We conducted a cross-sectional nationwide online survey in French pharmacy faculties to assess psychotropic drug use by pharmacy students from March 16, 2016 to May 20, 2016 (66 days). The internet address of the survey^1^ (Supplementary Data Sheet 1) was sent by email to all the French Pharmacy students (metropolitan France) through the student associations of the French pharmacy faculties and the national association of French pharmacy students [Association Nationale des Etudiants en Pharmacie de France (ANEPF)]. The online survey was also sent to pharmacy students through social networks (Facebook) and though toxicology teachers [Groupement Associé des Enseignants de Toxicologie (GATOX)]. The number of pharmacy students was estimated at 18,000 according to the national *numerus clausus* for the admission to French pharmacy studies in 2016–2017 (Journal Officiel de la République Française, 2017).

Participants could be included in the survey if they were 1st–6th year pharmacy students studying at a French pharmacy faculty. Exclusion criteria were defined as follows: other university course (different from pharmacy), pharmacy residents, young graduated pharmacists, age < 18 years.

The study was designed to conform to the STROBE (Strengthening the Reporting of Observational Studies in Epidemiology) guidelines for reporting observational studies (von Elm et al., 2007; Supplementary Table 2). The study was anonymous and registered with the local correspondent of the French Commission on Information Technology, Data Files and Civil Liberty (Commission Nationale de l’Informatique et des libertés, No.0160) in accordance with French law and approved by the local ethics committee (No. 2016/CE23, March 22, 2016, Comité de Protection des Personnes sud-est 6, IRB: 00008526). The participants’ consent was obtained with the answer to the survey.

### Survey Protocol

The primary outcome measured was psychotropic drug use (medications and illegal drugs) during the 3 last months (list of medications and illegal drugs, Supplementary Table 1), indication of use, type of prescription for medication (medical or self-medication) and frequency of use (*nota bene*: French pharmacists are not allowed to prescribe drugs). Secondary outcomes were the assessment of several comorbidities, such as anxiety and depression symptoms using the hospital anxiety and depression scale (HADS) at the time of the answer, considering the following thresholds, normal (total score ≤7), borderline or suggestive of possible anxiety/depression (total score of 8–10) and indicative of anxiety/depression (total score ≥11) (Zigmond and Snaith, 1983), stress of the last 7 days using a visual analogic scale (0–100 VAS: 0 no stress and 100 maximum stress imaginable) (Pottier et al., 2011) and fatigue of the last 7 days using a visual analogic scale (0–100 VAS: 0 no fatigue and 100 maximum fatigue imaginable) (Punja et al., 2014). Age and sex were recorded for the socio-demographic factors. Information on university studies was recorded with the present year of study, the annual average mark of last year (usually in France, marks are rated on a 20-point scale and assessed as follows: not validated <10/20, pass grade 10–12/20, fairly good grade 12–14/20, good grade 14–16/20 and very good grade >16/20), grade repetition, study difficulty perceived by the student (0–100 VAS score, 0: easy study and 100 very hard study) and attendance of the student (0–100 VAS score, 0: no attendance and 100: attendance to all the courses). Daily consumptions of cigarettes and electronic cigarettes, occasional alcohol consumption and the consumption of alcohol higher than the limits of 21 units per week for males and 14 units per weeks for females (defined as hazardous alcohol consumption) were also recorded. Approximately 20 min were required to complete the online survey.

Study data were collected and managed using REDCap electronic data capture tools hosted at CHU Clermont-Ferrand (Harris et al., 2009). REDCap (Research Electronic Data Capture) is a secure, web-based application designed to support data capture for research studies, providing: (1) an intuitive interface for validated data entry; (2) audit trails for tracking data manipulation and export procedures; (3) automated export procedures for seamless data downloads to common statistical packages; and (4) procedures for importing data from external sources.

Efforts have been done to address potential sources of bias. Selection bias should be limited because the survey has been sent to all the French Pharmacy students. However, there is no data available in France on age, sex and characteristics of these students, so it is difficult to ensure comparability. Reporting bias for this topic can be present and difficult to manage. For example, it is well known that alcohol consumption is frequently under-estimated by respondents (Stockwell et al., 2016). The quality of the questionnaire has been discussed and designed by a multidisciplinary group of pharmacist, addictologist, biostatistician, toxicologist, and epidemiologist.

### Statistical Considerations

Sample size was determined to achieve an accuracy of the confidence interval of prevalence of psychoactive drugs use greater than ± 3%. More precisely, it was necessary to include more than 1068 subjects, with such assumption, for a two-sided type I error at 5%.

Statistical analysis was performed using Stata 13 (StataCorp, College Station, TX, United States). The tests were two-sided, with a type-I error set at α = 0.05. Continuous data were expressed as mean ± standard deviation or median and interquartile range according to statistical distribution. The assumption of normality was studied using the Shapiro–Wilk test. The quantitative data were compared between independent groups using ANOVA or the Kruskal–Wallis test when the assumptions of ANOVA were not met. The assumption of homoscedasticity was studied using the Bartlett test. When appropriate (omnibus *p*-value < 0.05), *post hoc* tests for multiple comparisons were performed to take into account corrections of type I errors: Tukey–Kramer test after ANOVA or Dunn’s test post Kruskal–Wallis. The comparisons between groups concerning categorical data were performed using Chi-squared or Fisher’s exact tests followed when appropriate by Marascuilo’s procedure. Multivariable analyses were carried out to adjust results on the age and gender of pharmacy students using regression models (multiple linear for quantitative dependent outcomes and logistic for dichotomous). As less than 5% of data were missing, handling of missing data was not apply. However, sensitivity analyses were conducted to guaranty that these missing data did not influence the results (data not shown). As proposed by several statisticians, we chose to report all the individual *p*-values without applying any mathematical correction for distinct tests comparing groups. Specific attention was given to the magnitude of differences and to clinical relevance (Rothman, 1990; Feise, 2002).

## Results

### Sample Description

The survey lasting 66 days resulted in the collection of 2,609 answers of which 2,575 were assessable according to the inclusion/exclusion criteria. The description of the pharmacy students who answered the survey is presented in **Table 1**. According to the estimation of the number of pharmacy students in France (*N* = 18,000), the response rate was close to 14%.

**Table 1:** Characteristics of the pharmacy students at the time of responding to the survey.

Female (*N* = 2,575)	65.9%
Age (*N* = 2,575)	22.0 ± 2.3 Median: 22, Q1: 20, Q3: 23
Study year (*N* = 2,575)	
1st year	9.4%
2nd year	19.2%
3rd year	22.1%
4th year	17.9%
5th year	1.8%
6th year	9.6%
Annual average mark (*N* = 2,496)	
<10/20	5.7%
10–12/20	43.2%
12–14/20	36.9%
14–16/20	12.6%
>16/20	1.6%
Study difficulty (VAS score: 0–100) (*N* = 2,468)	69.2 ± 17.0 Median: 70, Q1:62, Q3: 80
Attendance (VAS score: 0–100) (*N* = 2,498)	58.5 ± 35.2 Median: 69, Q1:23, Q3: 93
Year repetition (*N* = 2,551)	40.9%
1st year repetition (*N* = 1,025)	66.3%
2nd year repetition (*N* = 1,025)	18.5%
3rd year repetition (*N* = 1,025)	9.0%
4th year repetition (*N* = 1,025)	3.1%
5th year repetition (*N* = 1,025)	2.7%
6th year repetition (*N* = 1,025)	0.3%
Tobacco consumption (daily) (*N* = 2,563)	18.7%
e-cigarette consumption (daily) (*N* = 2,549)	1.8%
Alcohol consumption (occasionally) (*N* = 2,156)	84.0%
Hazardous alcohol consumption, male (*N* = 768)	8.9%
Hazardous alcohol consumption, female (*N* = 1,388)	3.8%

### Use of Psychotropic Medications and Illegal Drugs

During the last 3 months, 9.4% (243/2,575) of respondents had used psychotropic medications [6.1% (157/2,575) only psychotropic medications], 21.5% (554/2,575) illegal drugs [18.2% (468/2,575) only illegal drugs] and 3.3% (86/2,575) both psychotropic medications and illegal drugs. It is important to note that 72.4% (1,864/2,575) of pharmacy students declared that they did not use any psychotropic medications or illegal drugs (defined in the study as non-users). Psychotropic medications were used in the cases of a medical prescription (49.0%, 119/243), a self-medication (42.4%, 103/243) or a non-medical intent (26.3%, 64/243).

Details of the psychotropic medications and illegal drugs used by the students during the last 3 months are presented in **Table 2**. Benzodiazepines and z drugs were the main psychotropic medications used (74.5%, 181/243) followed by opioid medications (29.2%, 71/243). Cannabis (cannabis herb and/or resin) was the main illegal drug used: 90.8% (503/554) of illegal drug users and 19.5% (503/2,575) of all the pharmacy students. MDMA use represented 3.5% of all the pharmacy students while cocaine was used by 2% (**Table 2**).

**Table 2:** List of the psychotropic medications and illegal drugs most used during the last 3 months.

Psychotropic medications (*N* = 243)		Illegal drugs (*N* = 554)	
Alprazolam	26.3%	Cannabis (herb)	82.7%
Bromazepam	17.7%	Cannabis (resin)	17.9%
Zolpidem	12.4%	MDMA	16.4%
Codeine	11.9%	Cocaine	9.4%
Tramadol	10.3%	Hallucinogenic mushroom	4.3%
Escitalopram	5.4%	LSD	2.9%
Zopiclone	5.4%	Cathinones	0%
Paroxetine	4.9%	Crack/free base	0%
Oxazepam	3.7%	Heroin	0%
Diazepam	3.3%	Methamphetamine	0%
Fluoxetine	2.1%	Synthetic cannabinoids	0%

*Only the main psychotropic medications are presented.*

Among benzodiazepines and z-drug users, 43.1% (78/181) were self-medicated and 40.3% (73/181) received a medical prescription. Non-medical use of benzodiazepines and Z-drugs was declared by 23.8% (43/181) of students using these medications. Among opioid users, 36.6% (26/71) were self-medicated and only 33.8% (24/71) had a medical prescription. Non-medical use of opioids was declared by 42.3% (30/71) of students using opioid medications.

The age of the students was significantly different between non-users, psychotropic medication users (medical prescription and self-medication) and illegal drug users (21.9 ± 2.3 vs. 22.8 ± 2.4 vs. 23.3 ± 2.4 vs. 22.0 ± 2.2, respectively, *p* < 0.001). Students using psychotropic medications were significantly older than non-users and illegal drug users (*p* < 0.05). Compared to non-users, the age difference was higher for alprazolam and bromazepam users (23.11 ± 2.48, *p* < 0.001) and for zolpidem users (23.27 ± 2.38, *p* = 0.001). When comparing the number of years of study, there was no difference between non-users and psychotropic medication (medical prescription) users. However, the proportions of self-medicated students were significantly different among the number of years of study and tended to increase over these years ([1st and 2nd year] 1.4% vs. [3rd and 4th year] 2.4% vs. [5th and 6th year] 4.4%, *p* = 0.001 and [1st and 2nd year] vs. [5th and 6th year], *p* < 0.05). Regarding illegal drugs, the proportions of users among the years of study were significantly different ([1st and 2nd year] 21.8% vs. [3rd and 4th year] 22.7% vs. [5th and 6th year] 18.1%, *p* = 0.047), but no significant difference stood out between years taken individually.

The proportion of females was different between non-users, psychotropic medication users (medical prescription and self-medication) and illegal drug users (73.1% vs. 62.2% vs. 64.3% vs. 43.7%, respectively, *p* < 0.001). Interestingly, although the population surveyed was predominantly female, there were significantly fewer females among the illegal drug users than non-users (*p* < 0.05), psychotropic medication users with medical prescriptions (*p* < 0.05) and psychotropic medication users with self-medication (*p* < 0.05).

**Table 3** shows the declared indications of the main psychotropic medications used (benzodiazepines: alprazolam and bromazepam; Z-drugs: zolpidem; opioids: codeine and tramadol). Self-medication with these psychotropic medications ranged between 28.0% (tramadol) and 51.7% (codeine). Zolpidem and bromazepam were both used for self-medication by half of the respondents. Depending on the psychotropic medication, non-medical use was declared by 21.9% (alprazolam) and 44.0% (tramadol) of users. Alprazolam and bromazepam were the most misused medications by pharmacy students (quantitatively).

**Table 3:** Indication, frequency of use of the main psychotropic self-medications.

	Alprazolam	Bromazepam	Zolpidem	Codeine	Tramadol
Medical prescription, *n*/*N* (%)	30/64 (46.9)	11/43 (25.6)	7/30 (23.3)	10/29 (34.5)	10/25 (40.0)
Self-medication, *n*/*N* (%)	24/64 (37.5)	22/43 (51.2)	15/30 (50.0)	15/29 (51.7)	7/25 (28.0)
Non-medical use, *n*/*N* (%)	14/64 (21.9)	13/43 (30.2)	9/30 (30.0)	9/29 (31.0)	11/25 (44.0)
Self-medication, *n* (%)	*N* = 24	*N* = 22	*N* = 15	*N* = 15	*N* = 7
Indications					
Anxiety (yes/no)	19 (79.2)	16 (72.7)	4 (26.7)	2 (13.3)	1 (14.3)
Insomnia (yes/no)	9 (37.5)	13 (59.1)	13 (86.7)	3 (20.0)	1 (14.3)
Depression (yes/no)	6 (25.0)	2 (9.1)	1 (6.7)	1 (6.7)	1 (14.3)
Pain (yes/no)	2 (8.3)	1 (4.6)	–	10 (66.7)	5 (71.4)
Frequency of use					
≥1/3 months	10 (41.7)	9 (40.9)	10 (66.7)	5 (33.3)	2 (28.6)
≥1/month	9 (37.5)	10 (45.5)	1 (6.7)	5 (33.3)	4 (57.1)
≥1/week	4 (20.8)	2 (9.1)	4 (26.7)	3 (26.7)	1 (14.3)
≥1/day	–	1 (4.6)	–	1 (6.7)	–

**Table 4** presents the declared objectives of use for the main illegal drugs: cannabis (herb), cannabis (resin), MDMA and cocaine.

**Table 4:** Indication and type of use of the main illegal drugs.

	Cannabis (herb)	Cannabis (resin)	MDMA	Cocaine
Indication, *n* (%)	*N* = 458	*N* = 99	*N* = 91	*N*= 52
Pleasure/recreational	421 (91.9)	88 (88.9)	88 (96.7)	47 (90.4)
Get stone	102 (22.3)	27 (27.3)	26 (28.6)	16 (30.8)
Self-medication	31 (6.8)	8 (8.1)	3 (3.3)	3 (5.8)
Self-medication indication, *n* (%)	*N* = 31	*N* = 8	*N* = 3	*N* = 3
Anxiety	15 (48.4)	5 (62.5)	1 (33.3)	1 (33.3)
Exam related stress	7 (22.6)	2 (25.0)	–	2 (66.7)
Sleep disturbances	20 (64.5)	6 (75.0)	2 (66.7)	2 (66.7)
Depression	14 (45.2)	3 (37.5)	1 (33.3)	1 (33.3)
Pain	11 (35.5)	–	–	1 (33.3)
Frequency of use, *n* (%)	*N* = 458	*N* = 99	*N* = 89	*N* = 51
≥1/3 months	227 (49.6)	43 (43.4)	65 (73.0)	41 (80.4)
≥1/month	91 (19.9)	21 (21.2)	21 (23.6)	7 (13.7)
≥1/week	102 (22.3)	23 (23.2)	3 (3.4)	3 (5.9)
≥1/day	38 (8.3)	12 (12.1)	–	–

*Among the 99 cannabis resin users, 54 also used cannabis herb.*

### Consequences of the Use of Psychotropic Medications and Illegal Drugs

The mean stress score of the last 7 days was 53.8 ± 23.9 for all the students. Stress scores were significantly higher for psychotropic medication users compared to non-users and illegal drug users (*p* < 0.05) (**Table 5**). This increase in the stress score was higher for alprazolam and/or bromazepam users than for non-users (66.7 ± 18.0 vs. 54.3 ± 23.3, *p* < 0.001).

**Table 5:** Use of psychotropic medications and illegal drugs and tobacco, alcohol use, stress, fatigue, annual average mark, attendance, study difficulty, and year repetition.

	Non-users	Psychotropic medications		Illegal drugs	*p*-values
		Medical prescription	Self-medication		
Tobacco, *n*/*N* (%)	199/1,856 (10.7)	8/81 (9.9)	9/70 (12.9)	253/533 (47.5)	<0.001^c,d,e^
e-cigarettes, *n*/*N* (%)	20/1,848 (1.1)	4/81 (4.9)	1/68 (1.5)	19/529 (3.6)	<0.001^c^
Alcohol, *n*/*N* (%)	1,494/1,860 (80.3)	66/82 (80.5)	54/69 (78.3)	519/534 (97.2)	<0.001^c,d,e^
Hazardous alcohol consumption, *n*/*N* (%)	41/1,494 (2.7)	2/66 (3.0)	1/54 (1.9)	69/519 (13.3)	<0.001^c,d,e^
Stress, mean ± sd	54.3 ± 23.3	62.3 ± 19.2	62.0 ± 19.2	49.9 ± 26.3	<0.001^a,b,c,d,e^
Fatigue, mean ± sd	63.0 ± 19.4	63.8 ± 21.9	66.4 ± 18.9	58.0 ± 22.3	<0.001^c,e^
Annual average mark, *n* (%)	*N* = 1,812	*N* = 77	*N* = 69	*N* = 516	
<12/20	844 (46.6)	39 (50.7)	29 (42.0)	294 (57.0)	<0.001^c^
12–14/20	683 (37.7)	31 (40.3)	28 (40.6)	173 (33.5)	NS
>14/20	285 (15.7)	7 (9.1)	12 (17.4)	49 (9.5)	<0.01^c^
Attendance, mean ± sd	64.8 ± 33.7	55.4 ± 35.3	54.6 ± 33.8	38.9 ± 32.7	<0.001^a,c,e^
Study difficulties, mean ± sd	70.6 ± 16.2	69.0 ± 16.1	68.3 ± 17.4	64.7 ± 19.1	<0.001^c^
Year repetition, *n*/*N* (%)	720/1,846 (39.0)	40/82 (48.8)	34/69 (49.3)	239/532 (44.9)	0.02

*^a^Non-users vs. psychotropic medications (medical prescription), p < 0.05. ^b^Non-users vs. psychotropic medications (self-medication), p < 0.05. ^c^Non-users vs. illegal drugs, p < 0.05. ^d^Psychotropic medications (medical prescription) vs. illegal drugs, ^e^Psychotropic medications (self-medication) vs. illegal drugs, p < 0.05.*

The mean fatigue score of the last 7 days was 62.0 ± 20.2 for all the students. Fatigue scores were significantly lower for illegal drug users compared to non-users and self-medicated students (*p* < 0.05) (**Table 5**). Self-medicated students had higher levels of fatigue than others but this difference was not significant.

Among all the pharmacy students, 18.7% (480/2,563) smoked tobacco, 1.8% (45/2,549) used e-cigarettes and 84.0% (2,156/2,568) drank alcoholic beverages [9.0% of males (68/754) and 4.0% of females (53/1,340) drank hazardous levels of alcohol]. Tobacco smoking and alcohol consumption (even hazardous alcohol consumption) were associated with illegal drug use (*p* < 0.001) (**Table 5**).

Annual average mark of the last year, attendance and perception of study difficulty were significantly lower for illegal drug users than for non-users (**Table 5**).

**FIGURE 1:**
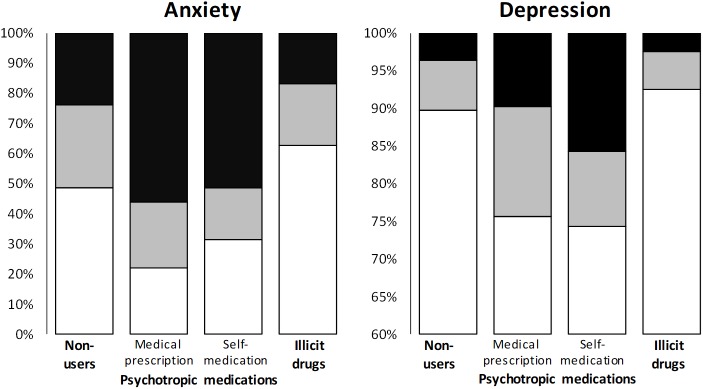
Association between use of psychotropic medications or illegal drugs and anxiety or depression. Scores of anxiety and depression were classified as normal (white), suggestive (gray) and indicative (black). Suggestive scores of anxiety: *p* = 0.003, *p* < 0.05: non-users vs. illegal drugs. Indicative scores of anxiety: *p* < 0.001, *p* < 0.05: non-users vs. medical prescription, *p* < 0.05: non-users vs. self-medication, *p* < 0.05: non-users vs. illegal drugs, *p* < 0.05: medical prescription vs. illegal drugs, *p* < 0.05: self-medication vs. illegal drugs. Suggestive scores of depression: *p* = 0.009, no difference between groups. Indicative scores of depression: *p* < 0.001, *p* < 0.05: self-medication vs. illegal drugs.

According to HADS scoring at the time of the answer, 25.5% (657/2,575) and 24.2% (624/2,575) of students had suggestive and indicative scores of anxiety, and 6.7% (173/2,575) and 3.9% (101/2,575) had suggestive and indicative scores of depression, respectively. Students taking psychotropic medications had higher levels of anxiety and depression than non-users and illegal drug users. Students self-medicated with psychotropic medications had even higher levels of anxiety and depression than students taking psychotropic medications with a medical prescription (**Figure 1**). Anxiety (indicative and suggestive HADS scores) affected 67.9% of psychotropic medication users (versus non-users, 47.9%, *p* < 0.001) and 78.0% of bromazepam and alprazolam users (versus non-users, *p* < 0.001). Similar trends were found for depression (indicative and suggestive HADS scores). Depression was found for 24.3% of psychotropic users (versus non-users, 9.2%, *p* < 0.001) and 33.0% of bromazepam and alprazolam users (versus non-users, *p* < 0.001). No difference for proportions of anxiety or depression stood out between zolpidem users and non-users.

Compared to non-users, illegal drug users had lower annual average marks (*p* < 0.01). Illegal drug users had also the lowest level of attendance compared to non-users and psychotropic medication users (*p* < 0.001). Finally and more surprisingly, illegal drug users felt that pharmacy studies were less difficult than non-users and psychotropic medication users (*p* < 0.001).

**FIGURE 2:**
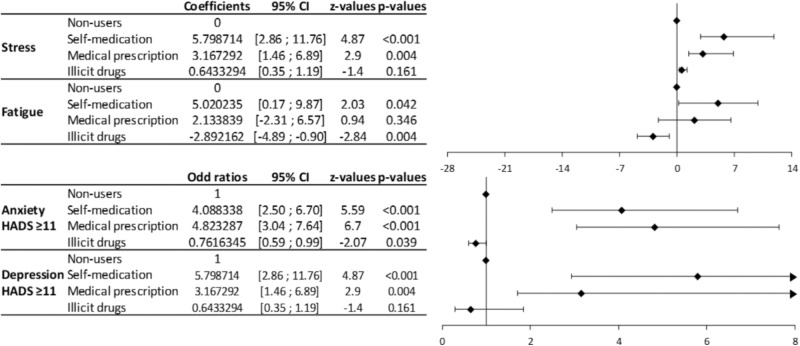
Forrest plot of the multivariate analyses of the relation between the use of psychotropic medications and illegal drugs with comorbidities. The multivariate analyses show coefficients for stress and fatigue scores (quantitative variables) and odd ratios for anxiety and depression (qualitative variables) related to the use of psychotropic medications and illegal drugs.

**FIGURE 3:**
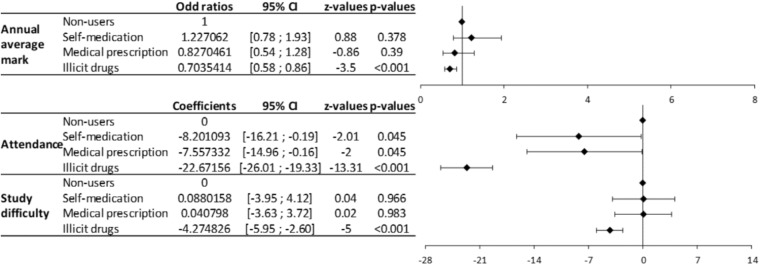
Forrest plot of the multivariate analyses of the relation between the use of psychotropic medications and illegal drugs with academic success. The multivariate analyses show coefficients for annual average mark (qualitative variables) and odd ratios for attendance and study difficulty (quantitative variables) related to the use of psychotropic medications and illegal drugs.

In the multivariate analysis, the students using psychotropic drugs (self-medication and medical prescription) presented significantly higher levels of stress, anxiety and depression compared to non-users (**Figure 2**). Conversely, the students using illegal drugs had lower levels of fatigue and anxiety compared to non-users (**Figure 2**). Students using illegal drugs had significantly lower annual average marks than non-users (**Figure 3**). Attendance was also significantly lower for students using psychotropic drugs or illegal drugs compared to non-users (*p* < 0.05), but the biggest decrease was for students using illegal drugs (*p* < 0.001). Perception of study difficulty was significantly lower for students using illegal drugs compared to non-users (*p* < 0.001) (**Figure 3**).

## Discussion

This study showed that among French pharmacy students, 9.4% used psychotropic medications during the last 3 months (benzodiazepines and Z-drugs: 7.0% of all pharmacy students; opioids: 2.7% of all pharmacy students), 21.5% used illegal drugs (19.5% of all pharmacy students used cannabis) and 3.3% both psychotropic medications and illegal drugs. These psychotropic medications were used in the case of a medical prescription by 49.0% of students, a self-medication by 42.4% and a non-medical intent by 26.3%. Stress scores of the last 7 days and proportions of anxiety and depression at the time of answer were higher for psychotropic medication users. Fatigue scores of the last 7 days and annual average marks of the last year, attendance and perception of study difficulty were lower for illegal drug users.

Uses of psychotropic medications had been assessed in 2000 for a smaller number of French students aged between 18 and 25 years old (*N* = 580), showing that 11.5% of students had used psychotropic medications in the past year (females: 21.0% and males: 6.4%) (Beck et al., 2005). In another large French study, the use of medically prescribed benzodiazepines was about 9.7% in 2013, 8.1% for anxiolytic benzodiazepines and 3.6% for hypnotic Z-drugs (18–44-years old, 69% women, *N* = 537,076) (Bénard-Laribière et al., 2016). In this last study, alprazolam, then zolpidem and bromazepam were the benzodiazepines most used (in our study by decreasing order: alprazolam, bromazepam, and zolpidem). Caution must be taken when interpreting these results because the age of the participants was not the same as those with Bénard-Laribière’s study, and benzodiazepine use increased with the age of the users (Bénard-Laribière et al., 2016; ANSM, 2017). The main mild opioid medications used in France in 2015 were tramadol, codeine combinations and opium combinations, while the strong opioids used were morphine (oral route) and oxycodone (with a very strong increase in use over the last few years +613%) (Hider-Mlynarz et al., 2018). In our study, codeine and tramadol were the main opioid drugs (1.1 and 1.0% of all pharmacy students).

For the Midwest United States college students from 2003 to 2013, between 21.7 and 25.8% had used in the past-year prescription medications (sleeping, sedative/anxiety, stimulant or pain medications) for medical use and 11.4–13.6% for non-medical use (McCabe et al., 2014). More precisely, 2.9–4.0% of these students had used sleeping medications for medical reasons (2.0–2.6% non-medically), 3.0–4.5% used sedative/anxiety medications for medical reasons (1.8–3.0% non-medically) and 15.7–21.4% used pain medications (opioids) for medical reasons (4.0–9.3% non-medically) (McCabe et al., 2014). The use of prescription opioids in Lebanese college students, in a weighted lifetime, was about 25% medically (medical prescription) and 15% non-medically (Ghandour et al., 2013). In comparison, French pharmacy students seemed to use quantitatively fewer psychotropic medications, but proportionally more sedative/anxiety medications and less opioid medications than students in other countries.

Self-medication with psychotropic medications was elevated (42.4%), even more elevated than medical prescriptions for bromazepam (51.2% vs. 25.6%), zolpidem (50.0% vs. 23.3%), and codeine (51.7% vs. 34.5%). This self-medication was mainly done following the authorized indications (i.e., anxiety and insomnia for benzodiazepines and Z-drugs; pain for opioids), but some respondents practiced self-medication without following these indications. For example, benzodiazepines were used for pain and opioids were used for anxiety, insomnia and depression. Moreover, we hypothesize that as students get older with their years of study and become more like “senior pharmacists,” they increase their self-medication with psychotropic medications, which is worrying since these behaviors may continue and increase over time. It is particularly worrying to see that students who self-medicated with psychotropic drugs had nearly the same disorders as students who received a medical prescription for psychotropic drugs. In this pharmacy student population, self-medication with psychotropic drugs seems to mask undiagnosed patients. This study highlighted the need for strengthening the screening of psychiatric disorders among pharmacy students and probably among all French students (e.g., medical, nurses, dentists, veterinarians…), in order to detect these undiagnosed patients and propose therapeutic care. This point has already been identified in the scientific literature and suggested that there are high levels of unmet need among University students (Fortney et al., 2016). Moreover, we recommend reinforcing information and preventive messages on psychotropic self-medication for pharmacy students and the necessity for these self-medicated students to see specialists (psychiatrists). This information could also be extended to other student groups (e.g., medical, nurses, dentists, veterinarians…) who might also have easy access to psychotropic drugs.

Self-medication with psychotropic medications, use of illegal drugs and alcohol abuse may also be associated with dual diagnosis, which is the co-occurrence of substance use disorders and mental disorders, well known among physicians (Braquehais et al., 2014). We hypothesize that these behaviors of self-medications with psychotropic medications, illegal drugs and alcohol abuse during university years may contribute to dual diagnosis in senior pharmacists.

Fortunately, both univariate and multivariate analyses did not show a strong negative impact of psychotropic medication use (medical prescription or self-medication) on academic achievement (annual average mark of the last year, repeating, study difficulties and attendance). These students could suffer from psychiatric (anxiety, depression), neurological (epilepsy, chronic pain) or rheumatological (chronic pain) diseases that may interfere negatively with their academic achievement due to disease relapses, the need to rest at home and hospitalizations.

In 2000 in France, 46.5% of students aged between 18 and 25 years old (*N* = 580) had experimented with cannabis at least once (males: 59.2% and females: 35.7%) and 29.7% at least once in the 12 past months (males: 38.3% and females: 22.3%) (Beck et al., 2005). In France in 2014, 17% of people aged between 18 and 25 years old had smoked cannabis during the last month, of whom 8% were regular users (at least 10 uses during the last 30 days) and 4% daily users (OFDT, 2017b). French pharmacy students have nearly the same cannabis consumption as the French population aged between 18 and 25 years old (19.5% for all pharmacy students during the last 3 months). Data from Ghandour et al. (2013) regarding a Lebanese college, showed that in a weighted lifetime 20.4% of students used cannabis herb, 2.8% MDMA and 1.6% cocaine/crack, which was very close to our study, in which 3.5% of French pharmacy students had used MDMA in the last 3 months and 2.0% cocaine.

The proportion of smokers and e-cigarette users seems to be low in French pharmacy students (18.7 and 1.8%, respectively). In 2016, 29.4% of French citizens aged 18–75 years smoked cigarettes daily and 2.5% e-cigarettes (OFDT, 2017c). About 21–23% of French students are daily smokers (Tavolacci et al., 2013; Gignon et al., 2015) while this figure is 22% for European students (Beck et al., 2014). The alcohol consumption of French pharmacy students was about 84.0% and hazardous alcohol consumption was 9.0% for males and 4.0% for females. Alcohol consumption by French college students is about 80.6–97% (Sommet et al., 2012; Gignon et al., 2015). This alcohol consumption was very close to French citizens aged 18–75 years of whom 87% had drunk alcohol at least once in the last 12 months in 2014 (OFDT, 2017a). In three American pharmacy colleges, 82.8% of students reported they had consumed alcohol in the past year and 25.4% consumed tobacco (Baldwin et al., 2011). In another American pharmacy college, about 86.5% of students had drunk alcohol during the past year and about 23.2% had hazardous or harmful alcohol use (AUDIT questionnaire ≥8/40) (Oliver et al., 2014). At the University of Leeds (United Kingdom), 86% of medical students declared they drank alcohol and 52.6% of the men and 50.6% of the women had a hazardous alcohol consumption (21 units for men and 14 units for women per week) (Pickard et al., 2000). In conclusion, the profile of French Pharmacy students regarding tobacco and alcohol consumption (20 and 85%, respectively) is very close to that of the French population in general, but they seem to have less hazardous alcohol consumption than Anglo-Saxon college students. Care is needed when comparing the results of the present study and those of the scientific literature because consumptions of tobacco, alcohol, psychotropic medications and illegal drugs are assessed differently regarding the quantities, frequencies, durations and years studied.

The misuse of psychotropic medications for non-medical use was also elevated and depended on the drug (alprazolam: 30.2%/0.5% and tramadol: 44.0%/0.4% for psychotropic medication users and for all pharmacy students, respectively). The psychiatric consequences associated with the use of psychotropic medications and illegal drugs have not been investigated in depth, but it is known that the non-medical use of prescription drugs (any, stimulant, sedative and opioid drugs) is significantly associated with psychiatric disorders and suicidal behavior among adolescents (Guo et al., 2016).

The other surprising result is that illegal drug users had lower levels of stress (during the last 7 days), fatigue (during the last 7 days), anxiety and depression (at the time on the answer) than non-users and users of psychotropic medications. However, illegal drug users had significantly higher tobacco and alcohol consumptions (even hazardous alcohol consumption). Illegal drug users had a very low level of attendance compared to non-users and users of psychotropic medications. These pharmacy students found their studies less difficult than did the other students. Year repetition was slightly more elevated in this population without significance, but the annual average mark was lower compared to non-users (57% of low annual average mark <12/20 and 9.5% Annual average mark >14/20).

Admittedly, French Pharmacy studies are difficult and exhausting for students. This point was related to the high level of anxiety (24.2% indicative score of HADS anxiety), which was nearly twice the prevalence of anxiety (11.7%) in college students from the WHO World Mental Health Surveys of college students from 21 countries and assessed with the Composite International Diagnostic Interview (Auerbach et al., 2016). This high level of anxiety was, however, close to the level of anxiety (21.6%, full Composite International Diagnostic Interview Short Form, CIDI-SF) found in French college students in 2005 (Kovess-Masfety et al., 2016) and found in medical, pharmacy and dentistry students from Lebanon (21.8% of severe and extremely severe anxiety assessed with the Depression Anxiety Stress Scales, DASS 21) (Younes et al., 2016). Levels of depression seemed to be lower for French Pharmacy students (3.9% indicative score of HADS for depression) compared to the WHO World Mental Health Surveys (6.0%, mood disorders) and the Lebanese students of medicine, pharmacy and dentistry (8%, severe and extremely severe depression, DASS21) (Younes et al., 2016), and French college students (8.5%, full CIDI-SF) (Kovess-Masfety et al., 2016).

Compared to a French sample of community pharmacists (*N* = 1,322 and age = 45.1 ± 10.6) studied for burnout and work-related stress in community pharmacies, senior pharmacists seemed to have a higher consumption of illicit drugs (24.5%) than pharmacy students (21.5%), whereas tobacco consumption (14.0%) and alcohol consumption (72.9%) tended to be lower than that of pharmacy students (18.7 and 84.0%, respectively) (Balayssac et al., 2017a). Scores of stress and fatigue remained very close between senior pharmacists and pharmacy students (stress: 52.31 ± 27.23 vs. 53.8 ± 23.9 and fatigue 59.6 ± 24.6 vs. 62.0 ± 20.2, respectively) (Balayssac et al., 2017b). However, proportions of indicative scores of anxiety and depression increased dramatically in senior pharmacists compared to students (42.4% vs. 24.2% and 15.7% vs. 3.9%, respectively) (Balayssac et al., 2017a).

### Study Limitations

The rate of response to the survey was low (14%). This low response rate could have been improved with a longer survey duration. Students of the 1st and the 6th year of pharmacy studies were less represented. During the 1st year of study, the students have a lot of work and competitive exams, so they would probably be less involved in “everyday life in the university.” Students of the 6th year performed an internship of 6 months (community pharmacy or pharmaceutical industry) during the survey schedule. Consequently, students from the 1st and the 6th years would be more difficult to recruit. Selection bias seems to be limited because psychotropic medication and illegal drug users did not seem to be over-represented in comparison to the scientific literature. It is also impossible to know if the population of the study is representative to the French pharmacy students, because there is no data available. The survey was sent by email to the students, which was a very good way to contact them, because they are all used to emails and digital environments. Each pharmacy faculty has digital work environment which allows students and professors to share lessons and homework. However, it was impossible to check that a student do not respond twice or more times.

## Conclusion

Regarding the main objective, we can conclude that the use of psychotropic medications and illegal drugs by French pharmacy students were similar to, but slightly lower than that of, the general French population. However, in comparison to other students in other countries, the use of psychotropic medications by French pharmacy students seemed to be lower, but with a proportionally higher use of anxiety/sedative medications and a lower use of opioid medications.

Besides the answer to the main objective and the prevalence of psychotropic medications and drug use by pharmacy students, two problems became clear. First, we think that pharmacy students using self-medication escape from a medical support and probably hide (consciously or unconsciously) their psychiatric disease. Second, illegal drugs have a negative impact on the academic success of pharmacy students. Other research could be engaged after this study. First, it would be very interesting to extend this study to medical students and to all students, in view to seeking an interaction between the type of study and the use of psychotropic medications and illegal drugs. Also, in the case of self-medication and non-medical use, we do not know how students get their medications: are they obtained through medical prescription or from pharmacy stocks? Therefore, it would be interesting to explore how pharmacy students obtain their medications. Academic doping could also be explored. Academic doping consists in taking medications or drugs in order to enhance cognitive capacities and academic success (Garasic and Lavazza, 2016). We can presume that pharmacy and medical studies, which are hard and selective, may be a strong determinant for academic doping.

## Author Contributions

DB, BP, MD, BV, and NA contributed to the design of the manuscript and the acquisition of data. LV and PC contributed to the acquisition of data. DB, BP, MD, LV, PC, AC, BV, and NA participated in drafting and revising the manuscript. All authors approved the final version of the manuscript for submission.

## Conflict of Interest Statement

The authors declare that the research was conducted in the absence of any commercial or financial relationships that could be construed as a potential conflict of interest.

## Footnotes

1 https://redcap.chu-clermontferrand.fr/surveys/?s=4YR8HMH8XM
