# Supplementary material for: Use of Psychotropic Medications and Illegal Drugs, and Related Consequences Among French Pharmacy Students – SCEP Study: A Nationwide Cross-Sectional Study
Source: Front Pharmacol. 2018 Jul 17;9:725. doi: 10.3389/fphar.2018.00725 (PMC6056660; doi:10.3389/fphar.2018.00725)
Supplement: Supplementary file 1 [file Table_1.pdf]

List of psychotropic medications

alprazolam (XANAX)
amisulpride (SOLIAN)
amitripyline (LAROXYL)
amoxapine (DEFANYL)
aripirazole (ABILIFY)
bromazepam (LEXOMIL)
carbamazepine (TEGRETOL)
chlorpromazine (LARGACTIL)
citalopram (SEROPRAM)
clobazam (URBANYL)
clomipramine (ANAFRANIL)
clonazepam (RIVOTRIL)
clorazepate dipotassique (TRANXENE)
clotiazepam (VERATRAN)
clozapine (LEPONEX)
codeine (CODOLIPRANE NEOCODION)
cyamemazine (TERCIAN)
dextrometorphan (TUXIUM)
diazepam (VALIUM)
dihydrocodeine (DICODIN)
divalproate de sodium (DEPAKOTE)
dosulepine (PROTHIADEN)
duloxetine (CYMBALTA)
escitalopram (SEROPLEX)
estazolam (NUCTALON)
fentanyl (ACTIQ, ABSTRAL)
flunitrazepam (ROHYPNOL)
fluoxetine (PROZAC)
flupentixol (FLUANXOL)
fluphenazine (MODITEN)
fluvoxamine (FLOXYFRAL)
haloperidol (HALDOL)
imipramine (TOFRANIL)
iproniazide (MARSILID)
levomepromazine (NOZINAN)
lithium (TERALITHE)
loprazolam (HAVLANE)
lorazepam (TEMESTA)
lormetazepam (NOCTAMIDE)
loxapine (LOXAPAC)
mianserine (ATHYMIL)
milnacipran (IXEL)
mirtazapine (NORSET)
moclobemide (MOCLAMINE)
morphine (SKENAN ACTISKENAN)
nitrazepam (MOGADON)
olanzapine (ZYPREXA)
oxazepam (SERESTA)
oxycodone (OXYCONTIN, OXYNORM)
paroxetine (DEROXAT)
pimozide (ORAP)
poudre d'opium (LAMALINE, IZALGI)
prazepam (LYSANXIA)
promethazine (PHENERGAN)
protoxyde d'azote (MEOPA)
risperidone (RISPERDAL)
sertraline (ZOLOFT)
sulpiride (DOGMATIL)
temazepam (NORMISON)
tramadol (TOPALGIC, CONTRAMAL)
triazolam (HALCION)
trimipramine (SURMONTIL)
valpromide (DEPAMIDE)
venlafaxine (EFFEXOR)
zolpidem (STILNOX)
zopiclone (IMOVANE)
OTHER

List of illegal psychotropic drugs

Amphetamine
Cannabis (herb)
Cannabis (resin)
Synthetic cannabinoids (spice)
Cathinones
Cocaine
Crack or free base
Ecstasy or MDMA
Hallucinogenic mushroom
Heroin
LSD
Methamphetamine
OTHER
