# Supplementary material for: Use of Psychotropic Medications and Illegal Drugs, and Related Consequences Among French Pharmacy Students – SCEP Study: A Nationwide Cross-Sectional Study
Source: Front Pharmacol. 2018 Jul 17;9:725. doi: 10.3389/fphar.2018.00725 (PMC6056660; doi:10.3389/fphar.2018.00725)
Supplement: Supplementary file 2 [file Table_2.pdf]

STROBE Statement—checklist of items that should be included in reports of observational studies

Use of psychotropic medications and illegal drugs, and related consequences among French pharmacy students – SCEP study: a nationwide cross-sectional study

	Item No	Recommendation	CHECK
**Title and abstract**	1	(a) Indicate the study's design with a commonly used term in the title or the abstract	See title<br>See abstract
		(b) Provide in the abstract an informative and balanced summary of what was done and what was found	See methods an results in abstract
**Introduction**			
Background/rationale	2	Explain the scientific background and rationale for the investigation being reported	See introduction<br>Line 64-130
Objectives	3	State specific objectives, including any prespecified hypotheses	Line 131-135
**Methods**			
Study design	4	Present key elements of study design early in the paper	See methods<br>1st section : study design
Setting	5	Describe the setting, locations, and relevant dates, including periods of recruitment, exposure, follow-up, and data collection	See methods<br>1st section : study design<br>1st paragraph
Participants	6	(a) Cohort study—Give the eligibility criteria, and the sources and methods of selection of participants. Describe methods of follow-up<br>Case-control study—Give the eligibility criteria, and the sources and methods of case ascertainment and control selection. Give the rationale for the choice of cases and controls<br>Cross-sectional study—Give the eligibility criteria, and the sources and methods of selection of participants	See methods<br>1st section : study design<br>2nd paragraph
		(b) Cohort study—For matched studies, give matching criteria and number of exposed and unexposed<br>Case-control study—For matched studies, give matching criteria and the number of controls per case	NA
Variables	7	Clearly define all outcomes, exposures, predictors, potential confounders, and effect modifiers. Give diagnostic criteria, if applicable	See methods<br>2nd section : Survey protocol
Data sources/ measurement	8*	For each variable of interest, give sources of data and details of methods of assessment (measurement). Describe comparability of assessment methods if there is more than one group	See methods<br>2nd section : Survey protocol
Bias	9	Describe any efforts to address potential sources of bias	See methods<br>2nd section : Survey protocol (last paragraph)
Study size	10	Explain how the study size was arrived at	See methods<br>3rd section : Statistical considerations
Quantitative variables	11	Explain how quantitative variables were handled in the analyses. If applicable, describe which groupings were chosen and why	See methods<br>3rd section : Statistical considerations
Statistical methods	12	(a) Describe all statistical methods, including those used to control for confounding	See methods<br>3rd section : Statistical considerations
		(b) Describe any methods used to examine subgroups and interactions	See methods<br>3rd section : Statistical considerations
		(c) Explain how missing data were addressed	See methods<br>3rd section : Statistical considerations
		(d) Cohort study—If applicable, explain how loss to follow-up was addressed	See methods

		Case-control study—If applicable, explain how matching of cases and controls was addressed<br>Cross-sectional study—If applicable, describe analytical methods taking account of sampling strategy	3rd section : Statistical considerations
		(e) Describe any sensitivity analyses	See methods<br>3rd section : Statistical considerations
**Results**			
Participants	13*	(a) Report numbers of individuals at each stage of study—eg numbers potentially eligible, examined for eligibility, confirmed eligible, included in the study, completing follow-up, and analysed	See results<br>Sample description and table 1
		(b) Give reasons for non-participation at each stage	See results<br>Sample description
		(c) Consider use of a flow diagram	It is no necessary<br>Only 34 students were exclude because of exclusion criteria<br><br>The number of included and exclude students are presented in the text (1st section of the results)
Descriptive data	14*	(a) Give characteristics of study participants (eg demographic, clinical, social) and information on exposures and potential confounders	See results<br>Sample description and table 1
		(b) Indicate number of participants with missing data for each variable of interest	These participants were exclude of the study because these variables remain in the inclusion and exclusion criteria
		(c) Cohort study—Summarise follow-up time (eg, average and total amount)	NA
Outcome data	15*	Cohort study—Report numbers of outcome events or summary measures over time	NA
		Case-control study—Report numbers in each exposure category, or summary measures of exposure	NA
		Cross-sectional study—Report numbers of outcome events or summary measures	See results and tables
Main results	16	(a) Give unadjusted estimates and, if applicable, confounder-adjusted estimates and their precision (eg, 95% confidence interval). Make clear which confounders were adjusted for and why they were included	See univariable and multiuvariable anaylsis
		(b) Report category boundaries when continuous variables were categorized	See results section and limit where explain
		(c) If relevant, consider translating estimates of relative risk into absolute risk for a meaningful time period	Please, see regression coefficient, odds ratio and 95%IC in the text and table/figures of the result section
Other analyses	17	Report other analyses done—eg analyses of subgroups and interactions, and sensitivity analyses	Please see analyses of subgroups (e.g. different user population …)
**Discussion**			
Key results	18	Summarise key results with reference to study objectives	See discussion 1st paragraph
Limitations	19	Discuss limitations of the study, taking into account sources of potential bias or imprecision. Discuss both direction and magnitude of any potential bias	See end of the discussion section
Interpretation	20	Give a cautious overall interpretation of results considering objectives, limitations, multiplicity of	See discussion

		analyses, results from similar studies, and other relevant evidence	
Generalisability	21	Discuss the generalisability (external validity) of the study results	See end of the discussion
**Other information**			
Funding	22	Give the source of funding and the role of the funders for the present study and, if applicable, for the original study on which the present article is based	This information has been sent to the Frontiers pharmacology web platform.

*Give information separately for cases and controls in case-control studies and, if applicable, for exposed and unexposed groups in cohort and cross-sectional studies.

Note: An Explanation and Elaboration article discusses each checklist item and gives methodological background and published examples of transparent reporting. The STROBE checklist is best used in conjunction with this article (freely available on the Web sites of PLoS Medicine at http://www.plosmedicine.org/, Annals of Internal Medicine at http://www.annals.org/, and Epidemiology at http://www.epidem.com/). Information on the STROBE Initiative is available at www.strobe-statement.org.
