# Supplementary material for: Use of Psychotropic Medications and Illegal Drugs, and Related Consequences Among French Pharmacy Students – SCEP Study: A Nationwide Cross-Sectional Study
Source: Front Pharmacol. 2018 Jul 17;9:725. doi: 10.3389/fphar.2018.00725 (PMC6056660; doi:10.3389/fphar.2018.00725)
Supplement: Supplementary file 3 [file Data_Sheet_1.PDF]

Evaluation de la consommation de substances psycho-actives par les étudiants des Facultés de pharmacie Française : Etude transversale nationale - SCEP

DANS LE CADRE D'UNE THESE DE PHARMACIE, LE LABORATOIRE DE TOXICOLOGIE DE LA FACULTE DE PHARMACIE DE CLERMONT-FERRAND EN PARTENARIAT AVEC LE CHU DE CLERMONT-FERRAND REALISENT UNE ENQUETE SUR LA CONSOMMATION DE SUBSTANCES PSYCHO-ACTIVES (MEDICAMENTS ET DROGUES) PAR LES ETUDIANTS DES FACULTES DE PHARMACIE.

CETTE ENQUETE EST TOTALEMENT ANONYME.

LA PARTICIPATION A CETTE ENQUETE EST LIBRE ET VOUS AVEZ LE CHOIX DE NE PAS PARTICIPER.

LA DUREE DU QUESTIONNAIRE EST D'ENVIRON 5 MIN MAXIMUM.

REPONDEZ LE PLUS SPONTANEMENT POSSIBLE AUX QUESTIONS POSEES.

MERCI DE NE REPONDRE QU'UNE SEULE FOIS A L'ENQUETE.

---

1. CONSOMMATION DE MEDICAMENTS PSYCHOTROPES

MERCI DE REPONDRE AUX QUESTIONS SUIVANTES

AVEZ-VOUS CONSOMME DANS LES 3 DERNIERS MOIS UN MEDICAMENT PSYCHO-ACTIF ? ○ OUI ○ NON

SI OUI, QUEL ETAIT CE MEDICAMENT :

◯ alprazolam (XANAX)
◯ amisulpride (SOLIAN)
◯ amitripyline (LAROXYL)
◯ amoxapine (DEFANYL)
◯ aripirazole (ABILIFY)
◯ bromazepam (LEXOMIL)
◯ carbamazepine (TEGRETOL)
◯ chlorpromazine (LARGACTIL)
◯ citalopram (SEROPRAM)
◯ clobazam (URBANYL)
◯ clomipramine (ANAFRANIL)
◯ clonazepam (RIVOTRIL)
◯ clorazepate dipotassique (TRANXENE)
◯ clotiazepam (VERATRAN)
◯ clozapine (LEPONEX)
◯ codeine (CODOLIPRANE NEOCODION)
◯ cyamemazine (TERCIAN)
◯ dextrometorphan (TUXIUM)
◯ diazepam (VALIUM)
◯ dihydrocodeine (DICODIN)
◯ divalproate de sodium (DEPAKOTE)
◯ dosulepine (PROTHIADEN)
◯ duloxetine (CYMBALTA)
◯ escitalopram (SEROPLEX)
◯ estazolam (NUCTALON)
◯ fentanyl (ACTIQ, ABSTRAL)
◯ flunitrazepam (ROHYPNOL)
◯ fluoxetine (PROZAC)
◯ flupentixol (FLUANXOL)
◯ fluphenazine (MODITEN)
◯ fluvoxamine (FLOXYFRAL)
◯ haloperidol (HALDOL)
◯ imipramine (TOFRANIL)
◯ iproniazide (MARSILID)
◯ levomepromazine (NOZINAN)
◯ lithium (TERALITHE)
◯ loprazolam (HAVLANE)
◯ lorazepam (TEMESTA)
◯ lormetazepam (NOCTAMIDE)
◯ loxapine (LOXAPAC)
◯ mianserine (ATHYMIL)
◯ milnacipran (IXEL)
◯ mirtazapine (NORSET)
◯ moclobemide (MOCLAMINE)
◯ morphine (SKENAN ACTISKENAN)
◯ nitrazepam (MOGADON)
◯ olanzapine (ZYPREXA)
◯ oxazepam (SERESTA)
◯ oxycodone (OXYCONTIN, OXYNORM)
◯ paroxetine (DEROXAT)
◯ pimozide (ORAP)
◯ poudre d'opium (LAMALINE, IZALGI)
◯ prazepam (LYSANXIA)
◯ promethazine (PHENERGAN)
◯ protoxyde d'azote (MEOPA)
◯ risperidone (RISPERDAL)
◯ sertraline (ZOLOFT)
◯ sulpiride (DOGMATIL)
◯ temazepam (NORMISON)
◯ tramadol (TOPALGIC, CONTRAMAL)
◯ triazolam (HALCION)
◯ trimipramine (SURMONTIL)
◯ valpromide (DEPAMIDE)
◯ venlafaxine (EFFEXOR)
◯ zolpidem (STILNOX)
◯ zopiclone (IMOVANE)
◯ AUTRE

SI AUTRE MEDICAMENT, LEQUEL : __________________________________

QUELLE A ETE LA FREQUENCE DE CETTE USAGE ?
- ◯ AU MOINS 1 FOIS DEPUIS 3 MOIS
- ◯ AU MOINS 1 FOIS PAR MOIS
- ◯ AU MOINS 1 FOIS PAR SEMAINE
- ◯ PLUSIEURS FOIS PAR SEMAINE
- ◯ TRAITEMENT OU USAGE QUOTIDIEN

DANS QUELLES CONDITIONS, CE MEDICAMENT A ETE UTILISE ?
- ☐ USAGE MEDICAL (PRESCRIPTION)
- ☐ USAGE MEDICAL (AUTOMEDICATION)
- ☐ USAGE NON MEDICAL (NON PRESCRIT)

QUELLE ETAIT L'INDICATION ?
- ☐ ANXIETE
- ☐ DEPRESSION
- ☐ INSOMNIE
- ☐ EPILEPSIE
- ☐ TROUBLES BIPOLAIRES
- ☐ DOULEUR
- ☐ USAGE RECREATIF
- ☐ DEFONCE
- ☐ AUTRE

SI AUTRE INDICATION, EXPLIQUER : __________________________________

AVEZ-VOUS CONSOMME DANS LES 3 DERNIERS MOIS UN AUTRE MEDICAMENT PSYCHO-ACTIF ?
◯ OUI ◯ NON

SI OUI, QUEL ETAIT CE MEDICAMENT :

- ◯ alprazolam (XANAX)
- ◯ amisulpride (SOLIAN)
- ◯ amitripyline (LAROXYL)
- ◯ amoxapine (DEFANYL)
- ◯ aripirazole (ABILIFY)
- ◯ bromazepam (LEXOMIL)
- ◯ carbamazepine (TEGRETOL)
- ◯ chlorpromazine (LARGACTIL)
- ◯ citalopram (SEROPRAM)
- ◯ clobazam (URBANYL)
- ◯ clomipramine (ANAFRANIL)
- ◯ clonazepam (RIVOTRIL)
- ◯ clorazepate dipotassique (TRANXENE)
- ◯ clotiazepam (VERATRAN)
- ◯ clozapine (LEPONEX)
- ◯ codeine (CODOLIPRANE NEOCODION)
- ◯ cyamemazine (TERCIAN)
- ◯ dextrometorphan (TUXIUM)
- ◯ diazepam (VALIUM)
- ◯ dihydrocodeine (DICODIN)
- ◯ divalproate de sodium (DEPAKOTE)
- ◯ dosulepine (PROTHIADEN)
- ◯ duloxetine (CYMBALTA)
- ◯ escitalopram (SEROPLEX)
- ◯ estazolam (NUCTALON)
- ◯ fentanyl (ACTIQ, ABSTRAL)
- ◯ flunitrazepam (ROHYPNOL)
- ◯ fluoxetine (PROZAC)
- ◯ flupentixol (FLUANXOL)
- ◯ fluphenazine (MODITEN)
- ◯ fluvoxamine (FLOXYFRAL)
- ◯ haloperidol (HALDOL)
- ◯ imipramine (TOFRANIL)
- ◯ iproniazide (MARSILID)
- ◯ levomepromazine (NOZINAN)
- ◯ lithium (TERALITHE)
- ◯ loprazolam (HAVLANE)
- ◯ lorazepam (TEMESTA)
- ◯ lormetazepam (NOCTAMIDE)
- ◯ loxapine (LOXAPAC)
- ◯ mianserine (ATHYMIL)
- ◯ milnacipran (IXEL)
- ◯ mirtazapine (NORSET)
- ◯ moclobemide (MOCLAMINE)
- ◯ morphine (SKENAN ACTISKENAN)
- ◯ nitrazepam (MOGADON)
- ◯ olanzapine (ZYPREXA)
- ◯ oxazepam (SERESTA)
- ◯ oxycodone (OXYCONTIN, OXYNORM)
- ◯ paroxetine (DEROXAT)
- ◯ pimozide (ORAP)
- ◯ poudre d'opium (LAMALINE, IZALGI)
- ◯ prazepam (LYSANXIA)
- ◯ promethazine (PHENERGAN)
- ◯ protoxyde d'azote (MEOPA)
- ◯ risperidone (RISPERDAL)
- ◯ sertraline (ZOLOFT)
- ◯ sulpiride (DOGMATIL)
- ◯ temazepam (NORMISON)
- ◯ tramadol (TOPALGIC, CONTRAMAL)
- ◯ triazolam (HALCION)
- ◯ trimipramine (SURMONTIL)
- ◯ valpromide (DEPAMIDE)
- ◯ venlafaxine (EFFEXOR)
- ◯ zolpidem (STILNOX)
- ◯ zopiclone (IMOVANE)
- ◯ AUTRE

SI AUTRE MEDICAMENT, LEQUEL : ______________________

QUELLE A ETE LA FREQUENCE DE CETTE USAGE ?
- ◯ AU MOINS 1 FOIS DEPUIS 3 MOIS
- ◯ AU MOINS 1 FOIS PAR MOIS
- ◯ AU MOINS 1 FOIS PAR SEMAINE
- ◯ PLUSIEURS FOIS PAR SEMAINE
- ◯ TRAITEMENT OU USAGE QUOTIDIEN

DANS QUELLES CONDITIONS, CE MEDICAMENT A ETE UTILISE ?
- ☐ USAGE MEDICAL (PRESCRIPTION)
- ☐ USAGE MEDICAL (AUTOMEDICATION)
- ☐ USAGE NON MEDICAL (NON PRESCRIT)

QUELLE ETAIT L'INDICATION ?
- ☐ ANXIETE
- ☐ DEPRESSION
- ☐ INSOMNIE
- ☐ EPILEPSIE
- ☐ TROUBLES BIPOLAIRES
- ☐ DOULEUR
- ☐ USAGE RECREATIF
- ☐ DEFONCE
- ☐ AUTRE

SI AUTRE INDICATION, EXPLIQUER : ______________________

AVEZ-VOUS CONSOMME DANS LES 3 DERNIERS MOIS UN AUTRE MEDICAMENT PSYCHO-ACTIF ?
◯ OUI ◯ NON

SI OUI, QUEL ETAIT CE MEDICAMENT :

◯ alprazolam (XANAX)
◯ amisulpride (SOLIAN)
◯ amitripyline (LAROXYL)
◯ amoxapine (DEFANYL)
◯ aripirazole (ABILIFY)
◯ bromazepam (LEXOMIL)
◯ carbamazepine (TEGRETOL)
◯ chlorpromazine (LARGACTIL)
◯ citalopram (SEROPRAM)
◯ clobazam (URBANYL)
◯ clomipramine (ANAFRANIL)
◯ clonazepam (RIVOTRIL)
◯ clorazepate dipotassique (TRANXENE)
◯ clotiazepam (VERATRAN)
◯ clozapine (LEPONEX)
◯ codeine (CODOLIPRANE NEOCODION)
◯ cyamemazine (TERCIAN)
◯ dextrometorphan (TUXIUM)
◯ diazepam (VALIUM)
◯ dihydrocodeine (DICODIN)
◯ divalproate de sodium (DEPAKOTE)
◯ dosulepine (PROTHIADEN)
◯ duloxetine (CYMBALTA)
◯ escitalopram (SEROPLEX)
◯ estazolam (NUCTALON)
◯ fentanyl (ACTIQ, ABSTRAL)
◯ flunitrazepam (ROHYPNOL)
◯ fluoxetine (PROZAC)
◯ flupentixol (FLUANXOL)
◯ fluphenazine (MODITEN)
◯ fluvoxamine (FLOXYFRAL)
◯ haloperidol (HALDOL)
◯ imipramine (TOFRANIL)
◯ iproniazide (MARSILID)
◯ levomepromazine (NOZINAN)
◯ lithium (TERALITHE)
◯ loprazolam (HAVLANE)
◯ lorazepam (TEMESTA)
◯ lormetazepam (NOCTAMIDE)
◯ loxapine (LOXAPAC)
◯ mianserine (ATHYMIL)
◯ milnacipran (IXEL)
◯ mirtazapine (NORSET)
◯ moclobemide (MOCLAMINE)
◯ morphine (SKENAN ACTISKENAN)
◯ nitrazepam (MOGADON)
◯ olanzapine (ZYPREXA)
◯ oxazepam (SERESTA)
◯ oxycodone (OXYCONTIN, OXYNORM)
◯ paroxetine (DEROXAT)
◯ pimozide (ORAP)
◯ poudre d'opium (LAMALINE, IZALGI)
◯ prazepam (LYSANXIA)
◯ promethazine (PHENERGAN)
◯ protoxyde d'azote (MEOPA)
◯ risperidone (RISPERDAL)
◯ sertraline (ZOLOFT)
◯ sulpiride (DOGMATIL)
◯ temazepam (NORMISON)
◯ tramadol (TOPALGIC, CONTRAMAL)
◯ triazolam (HALCION)
◯ trimipramine (SURMONTIL)
◯ valpromide (DEPAMIDE)
◯ venlafaxine (EFFEXOR)
◯ zolpidem (STILNOX)
◯ zopiclone (IMOVANE)
◯ AUTRE

SI AUTRE MEDICAMENT, LEQUEL : __________________________________

QUELLE A ETE LA FREQUENCE DE CETTE USAGE ?
- ◯ AU MOINS 1 FOIS DEPUIS 3 MOIS
- ◯ AU MOINS 1 FOIS PAR MOIS
- ◯ AU MOINS 1 FOIS PAR SEMAINE
- ◯ PLUSIEURS FOIS PAR SEMAINE
- ◯ TRAITEMENT OU USAGE QUOTIDIEN

DANS QUELLES CONDITIONS, CE MEDICAMENT A ETE UTILISE ?
- ☐ USAGE MEDICAL (PRESCRIPTION)
- ☐ USAGE MEDICAL (AUTOMEDICATION)
- ☐ USAGE NON MEDICAL (NON PRESCRIT)

QUELLE ETAIT L'INDICATION ?
- ☐ ANXIETE
- ☐ DEPRESSION
- ☐ INSOMNIE
- ☐ EPILEPSIE
- ☐ TROUBLES BIPOLAIRES
- ☐ DOULEUR
- ☐ USAGE RECREATIF
- ☐ DEFONCE
- ☐ AUTRE

SI AUTRE INDICATION, EXPLIQUER : __________________________________

AVEZ-VOUS CONSOMME DANS LES 3 DERNIERS MOIS UN AUTRE MEDICAMENT PSYCHO-ACTIF ?
◯ OUI ◯ NON

SI OUI, QUEL ETAIT CE MEDICAMENT :

- ◯ alprazolam (XANAX)
- ◯ amisulpride (SOLIAN)
- ◯ amitripyline (LAROXYL)
- ◯ amoxapine (DEFANYL)
- ◯ aripirazole (ABILIFY)
- ◯ bromazepam (LEXOMIL)
- ◯ carbamazepine (TEGRETOL)
- ◯ chlorpromazine (LARGACTIL)
- ◯ citalopram (SEROPRAM)
- ◯ clobazam (URBANYL)
- ◯ clomipramine (ANAFRANIL)
- ◯ clonazepam (RIVOTRIL)
- ◯ clorazepate dipotassique (TRANXENE)
- ◯ clotiazepam (VERATRAN)
- ◯ clozapine (LEPONEX)
- ◯ codeine (CODOLIPRANE NEOCODION)
- ◯ cyamemazine (TERCIAN)
- ◯ dextrometorphan (TUXIUM)
- ◯ diazepam (VALIUM)
- ◯ dihydrocodeine (DICODIN)
- ◯ divalproate de sodium (DEPAKOTE)
- ◯ dosulepine (PROTHIADEN)
- ◯ duloxetine (CYMBALTA)
- ◯ escitalopram (SEROPLEX)
- ◯ estazolam (NUCTALON)
- ◯ fentanyl (ACTIQ, ABSTRAL)
- ◯ flunitrazepam (ROHYPNOL)
- ◯ fluoxetine (PROZAC)
- ◯ flupentixol (FLUANXOL)
- ◯ fluphenazine (MODITEN)
- ◯ fluvoxamine (FLOXYFRAL)
- ◯ haloperidol (HALDOL)
- ◯ imipramine (TOFRANIL)
- ◯ iproniazide (MARSILID)
- ◯ levomepromazine (NOZINAN)
- ◯ lithium (TERALITHE)
- ◯ loprazolam (HAVLANE)
- ◯ lorazepam (TEMESTA)
- ◯ lormetazepam (NOCTAMIDE)
- ◯ loxapine (LOXAPAC)
- ◯ mianserine (ATHYMIL)
- ◯ milnacipran (IXEL)
- ◯ mirtazapine (NORSET)
- ◯ moclobemide (MOCLAMINE)
- ◯ morphine (SKENAN ACTISKENAN)
- ◯ nitrazepam (MOGADON)
- ◯ olanzapine (ZYPREXA)
- ◯ oxazepam (SERESTA)
- ◯ oxycodone (OXYCONTIN, OXYNORM)
- ◯ paroxetine (DEROXAT)
- ◯ pimozide (ORAP)
- ◯ poudre d'opium (LAMALINE, IZALGI)
- ◯ prazepam (LYSANXIA)
- ◯ promethazine (PHENERGAN)
- ◯ protoxyde d'azote (MEOPA)
- ◯ risperidone (RISPERDAL)
- ◯ sertraline (ZOLOFT)
- ◯ sulpiride (DOGMATIL)
- ◯ temazepam (NORMISON)
- ◯ tramadol (TOPALGIC, CONTRAMAL)
- ◯ triazolam (HALCION)
- ◯ trimipramine (SURMONTIL)
- ◯ valpromide (DEPAMIDE)
- ◯ venlafaxine (EFFEXOR)
- ◯ zolpidem (STILNOX)
- ◯ zopiclone (IMOVANE)
- ◯ AUTRE

SI AUTRE MEDICAMENT, LEQUEL : ______________________

QUELLE A ETE LA FREQUENCE DE CETTE USAGE ?
- ◯ AU MOINS 1 FOIS DEPUIS 3 MOIS
- ◯ AU MOINS 1 FOIS PAR MOIS
- ◯ AU MOINS 1 FOIS PAR SEMAINE
- ◯ PLUSIEURS FOIS PAR SEMAINE
- ◯ TRAITEMENT OU USAGE QUOTIDIEN

DANS QUELLES CONDITIONS, CE MEDICAMENT A ETE UTILISE ?
- ☐ USAGE MEDICAL (PRESCRIPTION)
- ☐ USAGE MEDICAL (AUTOMEDICATION)
- ☐ USAGE NON MEDICAL (NON PRESCRIT)

QUELLE ETAIT L'INDICATION ?
- ☐ ANXIETE
- ☐ DEPRESSION
- ☐ INSOMNIE
- ☐ EPILEPSIE
- ☐ TROUBLES BIPOLAIRES
- ☐ DOULEUR
- ☐ USAGE RECREATIF
- ☐ DEFONCE
- ☐ AUTRE

SI AUTRE INDICATION, EXPLIQUER : ______________________

AVEZ-VOUS CONSOMME DANS LES 3 DERNIERS MOIS UN AUTRE MEDICAMENT PSYCHO-ACTIF ? ◯ OUI ◯ NON

SI OUI, QUEL ETAIT CE MEDICAMENT :

- ◯ alprazolam (XANAX)
- ◯ amisulpride (SOLIAN)
- ◯ amitripyline (LAROXYL)
- ◯ amoxapine (DEFANYL)
- ◯ aripirazole (ABILIFY)
- ◯ bromazepam (LEXOMIL)
- ◯ carbamazepine (TEGRETOL)
- ◯ chlorpromazine (LARGACTIL)
- ◯ citalopram (SEROPRAM)
- ◯ clobazam (URBANYL)
- ◯ clomipramine (ANAFRANIL)
- ◯ clonazepam (RIVOTRIL)
- ◯ clorazepate dipotassique (TRANXENE)
- ◯ clotiazepam (VERATRAN)
- ◯ clozapine (LEPONEX)
- ◯ codeine (CODOLIPRANE NEOCODION)
- ◯ cyamemazine (TERCIAN)
- ◯ dextrometorphan (TUXIUM)
- ◯ diazepam (VALIUM)
- ◯ dihydrocodeine (DICODIN)
- ◯ divalproate de sodium (DEPAKOTE)
- ◯ dosulepine (PROTHIADEN)
- ◯ duloxetine (CYMBALTA)
- ◯ escitalopram (SEROPLEX)
- ◯ estazolam (NUCTALON)
- ◯ fentanyl (ACTIQ, ABSTRAL)
- ◯ flunitrazepam (ROHYPNOL)
- ◯ fluoxetine (PROZAC)
- ◯ flupentixol (FLUANXOL)
- ◯ fluphenazine (MODITEN)
- ◯ fluvoxamine (FLOXYFRAL)
- ◯ haloperidol (HALDOL)
- ◯ imipramine (TOFRANIL)
- ◯ iproniazide (MARSILID)
- ◯ levomepromazine (NOZINAN)
- ◯ lithium (TERALITHE)
- ◯ loprazolam (HAVLANE)
- ◯ lorazepam (TEMESTA)
- ◯ lormetazepam (NOCTAMIDE)
- ◯ loxapine (LOXAPAC)
- ◯ mianserine (ATHYMIL)
- ◯ milnacipran (IXEL)
- ◯ mirtazapine (NORSET)
- ◯ moclobemide (MOCLAMINE)
- ◯ morphine (SKENAN ACTISKENAN)
- ◯ nitrazepam (MOGADON)
- ◯ olanzapine (ZYPREXA)
- ◯ oxazepam (SERESTA)
- ◯ oxycodone (OXYCONTIN, OXYNORM)
- ◯ paroxetine (DEROXAT)
- ◯ pimozide (ORAP)
- ◯ poudre d'opium (LAMALINE, IZALGI)
- ◯ prazepam (LYSANXIA)
- ◯ promethazine (PHENERGAN)
- ◯ protoxyde d'azote (MEOPA)
- ◯ risperidone (RISPERDAL)
- ◯ sertraline (ZOLOFT)
- ◯ sulpiride (DOGMATIL)
- ◯ temazepam (NORMISON)
- ◯ tramadol (TOPALGIC, CONTRAMAL)
- ◯ triazolam (HALCION)
- ◯ trimipramine (SURMONTIL)
- ◯ valpromide (DEPAMIDE)
- ◯ venlafaxine (EFFEXOR)
- ◯ zolpidem (STILNOX)
- ◯ zopiclone (IMOVANE)
- ◯ AUTRE

SI AUTRE MEDICAMENT, LEQUEL : __________________

QUELLE A ETE LA FREQUENCE DE CETTE USAGE ?
- ○ AU MOINS 1 FOIS DEPUIS 3 MOIS
- ○ AU MOINS 1 FOIS PAR MOIS
- ○ AU MOINS 1 FOIS PAR SEMAINE
- ○ PLUSIEURS FOIS PAR SEMAINE
- ○ TRAITEMENT OU USAGE QUOTIDIEN

DANS QUELLES CONDITIONS, CE MEDICAMENT A ETE UTILISE ?
- ☐ USAGE MEDICAL (PRESCRIPTION)
- ☐ USAGE MEDICAL (AUTOMEDICATION)
- ☐ USAGE NON MEDICAL (NON PRESCRIT)

QUELLE ETAIT L'INDICATION ?
- ☐ ANXIETE
- ☐ DEPRESSION
- ☐ INSOMNIE
- ☐ EPILEPSIE
- ☐ TROUBLES BIPOLAIRES
- ☐ DOULEUR
- ☐ USAGE RECREATIF
- ☐ DEFONCE
- ☐ AUTRE

SI AUTRE INDICATION, EXPLIQUER : __________________

AVEZ-VOUS CONSOMME DANS LES 3 DERNIERS MOIS UN AUTRE MEDICAMENT PSYCHO-ACTIF ? ○ OUI ○ NON

SI OUI, QUEL ETAIT CE MEDICAMENT :

◯ alprazolam (XANAX)
◯ amisulpride (SOLIAN)
◯ amitripyline (LAROXYL)
◯ amoxapine (DEFANYL)
◯ aripirazole (ABILIFY)
◯ bromazepam (LEXOMIL)
◯ carbamazepine (TEGRETOL)
◯ chlorpromazine (LARGACTIL)
◯ citalopram (SEROPRAM)
◯ clobazam (URBANYL)
◯ clomipramine (ANAFRANIL)
◯ clonazepam (RIVOTRIL)
◯ clorazepate dipotassique (TRANXENE)
◯ clotiazepam (VERATRAN)
◯ clozapine (LEPONEX)
◯ codeine (CODOLIPRANE NEOCODION)
◯ cyamemazine (TERCIAN)
◯ dextrometorphan (TUXIUM)
◯ diazepam (VALIUM)
◯ dihydrocodeine (DICODIN)
◯ divalproate de sodium (DEPAKOTE)
◯ dosulepine (PROTHIADEN)
◯ duloxetine (CYMBALTA)
◯ escitalopram (SEROPLEX)
◯ estazolam (NUCTALON)
◯ fentanyl (ACTIQ, ABSTRAL)
◯ flunitrazepam (ROHYPNOL)
◯ fluoxetine (PROZAC)
◯ flupentixol (FLUANXOL)
◯ fluphenazine (MODITEN)
◯ fluvoxamine (FLOXYFRAL)
◯ haloperidol (HALDOL)
◯ imipramine (TOFRANIL)
◯ iproniazide (MARSILID)
◯ levomepromazine (NOZINAN)
◯ lithium (TERALITHE)
◯ loprazolam (HAVLANE)
◯ lorazepam (TEMESTA)
◯ lormetazepam (NOCTAMIDE)
◯ loxapine (LOXAPAC)
◯ mianserine (ATHYMIL)
◯ milnacipran (IXEL)
◯ mirtazapine (NORSET)
◯ moclobemide (MOCLAMINE)
◯ morphine (SKENAN ACTISKENAN)
◯ nitrazepam (MOGADON)
◯ olanzapine (ZYPREXA)
◯ oxazepam (SERESTA)
◯ oxycodone (OXYCONTIN, OXYNORM)
◯ paroxetine (DEROXAT)
◯ pimozide (ORAP)
◯ poudre d'opium (LAMALINE, IZALGI)
◯ prazepam (LYSANXIA)
◯ promethazine (PHENERGAN)
◯ protoxyde d'azote (MEOPA)
◯ risperidone (RISPERDAL)
◯ sertraline (ZOLOFT)
◯ sulpiride (DOGMATIL)
◯ temazepam (NORMISON)
◯ tramadol (TOPALGIC, CONTRAMAL)
◯ triazolam (HALCION)
◯ trimipramine (SURMONTIL)
◯ valpromide (DEPAMIDE)
◯ venlafaxine (EFFEXOR)
◯ zolpidem (STILNOX)
◯ zopiclone (IMOVANE)
◯ AUTRE

SI AUTRE MEDICAMENT, LEQUEL : ______________________

QUELLE A ETE LA FREQUENCE DE CETTE USAGE ?
- ◯ AU MOINS 1 FOIS DEPUIS 3 MOIS
- ◯ AU MOINS 1 FOIS PAR MOIS
- ◯ AU MOINS 1 FOIS PAR SEMAINE
- ◯ PLUSIEURS FOIS PAR SEMAINE
- ◯ TRAITEMENT OU USAGE QUOTIDIEN

DANS QUELLES CONDITIONS, CE MEDICAMENT A ETE UTILISE ?
- ☐ USAGE MEDICAL (PRESCRIPTION)
- ☐ USAGE MEDICAL (AUTOMEDICATION)
- ☐ USAGE NON MEDICAL (NON PRESCRIT)

QUELLE ETAIT L'INDICATION ?
- ☐ ANXIETE
- ☐ DEPRESSION
- ☐ INSOMNIE
- ☐ EPILEPSIE
- ☐ TROUBLES BIPOLAIRES
- ☐ DOULEUR
- ☐ USAGE RECREATIF
- ☐ DEFONCE
- ☐ AUTRE

SI AUTRE INDICATION, EXPLIQUER : ______________________

AVEZ-VOUS CONSOMME DANS LES 3 DERNIERS MOIS UN AUTRE MEDICAMENT PSYCHO-ACTIF ? ◯ OUI ◯ NON

SI OUI, QUEL ETAIT CE MEDICAMENT :

◯ alprazolam (XANAX)
◯ amisulpride (SOLIAN)
◯ amitripyline (LAROXYL)
◯ amoxapine (DEFANYL)
◯ aripirazole (ABILIFY)
◯ bromazepam (LEXOMIL)
◯ carbamazepine (TEGRETOL)
◯ chlorpromazine (LARGACTIL)
◯ citalopram (SEROPRAM)
◯ clobazam (URBANYL)
◯ clomipramine (ANAFRANIL)
◯ clonazepam (RIVOTRIL)
◯ clorazepate dipotassique (TRANXENE)
◯ clotiazepam (VERATRAN)
◯ clozapine (LEPONEX)
◯ codeine (CODOLIPRANE NEOCODION)
◯ cyamemazine (TERCIAN)
◯ dextrometorphan (TUXIUM)
◯ diazepam (VALIUM)
◯ dihydrocodeine (DICODIN)
◯ divalproate de sodium (DEPAKOTE)
◯ dosulepine (PROTHIADEN)
◯ duloxetine (CYMBALTA)
◯ escitalopram (SEROPLEX)
◯ estazolam (NUCTALON)
◯ fentanyl (ACTIQ, ABSTRAL)
◯ flunitrazepam (ROHYPNOL)
◯ fluoxetine (PROZAC)
◯ flupentixol (FLUANXOL)
◯ fluphenazine (MODITEN)
◯ fluvoxamine (FLOXYFRAL)
◯ haloperidol (HALDOL)
◯ imipramine (TOFRANIL)
◯ iproniazide (MARSILID)
◯ levomepromazine (NOZINAN)
◯ lithium (TERALITHE)
◯ loprazolam (HAVLANE)
◯ lorazepam (TEMESTA)
◯ lormetazepam (NOCTAMIDE)
◯ loxapine (LOXAPAC)
◯ mianserine (ATHYMIL)
◯ milnacipran (IXEL)
◯ mirtazapine (NORSET)
◯ moclobemide (MOCLAMINE)
◯ morphine (SKENAN ACTISKENAN)
◯ nitrazepam (MOGADON)
◯ olanzapine (ZYPREXA)
◯ oxazepam (SERESTA)
◯ oxycodone (OXYCONTIN, OXYNORM)
◯ paroxetine (DEROXAT)
◯ pimozide (ORAP)
◯ poudre d'opium (LAMALINE, IZALGI)
◯ prazepam (LYSANXIA)
◯ promethazine (PHENERGAN)
◯ protoxyde d'azote (MEOPA)
◯ risperidone (RISPERDAL)
◯ sertraline (ZOLOFT)
◯ sulpiride (DOGMATIL)
◯ temazepam (NORMISON)
◯ tramadol (TOPALGIC, CONTRAMAL)
◯ triazolam (HALCION)
◯ trimipramine (SURMONTIL)
◯ valpromide (DEPAMIDE)
◯ venlafaxine (EFFEXOR)
◯ zolpidem (STILNOX)
◯ zopiclone (IMOVANE)
◯ AUTRE

SI AUTRE MEDICAMENT, LEQUEL : ______________________

QUELLE A ETE LA FREQUENCE DE CETTE USAGE ?
- ○ AU MOINS 1 FOIS DEPUIS 3 MOIS
- ○ AU MOINS 1 FOIS PAR MOIS
- ○ AU MOINS 1 FOIS PAR SEMAINE
- ○ PLUSIEURS FOIS PAR SEMAINE
- ○ TRAITEMENT OU USAGE QUOTIDIEN

DANS QUELLES CONDITIONS, CE MEDICAMENT A ETE UTILISE ?
- ☐ USAGE MEDICAL (PRESCRIPTION)
- ☐ USAGE MEDICAL (AUTOMEDICATION)
- ☐ USAGE NON MEDICAL (NON PRESCRIT)

QUELLE ETAIT L'INDICATION ?
- ☐ ANXIETE
- ☐ DEPRESSION
- ☐ INSOMNIE
- ☐ EPILEPSIE
- ☐ TROUBLES BIPOLAIRES
- ☐ DOULEUR
- ☐ USAGE RECREATIF
- ☐ DEFONCE
- ☐ AUTRE

SI AUTRE INDICATION, EXPLIQUER : ______________________

---

2. CONSOMMATION DE PRODUITS STUPEFIANTS

MERCI DE REPONDRE AUX QUESTIONS SUIVANTES

AVEZ-VOUS CONSOMME DANS LES 3 DERNIERS MOIS UNE SUBSTANCE STUPEFIANTE ?
○ OUI ○ NON

SI OUI, LAQUELLE ?
- ○ AMPHETAMINE
- ○ CANNABIS (HERBE)
- ○ CANNABIS (RESINE)
- ○ CANNABINOIDES DE SYNTHESE (SPICE)
- ○ CATHINONES
- ○ CHAMPIGNONS HALLUCINOGENES
- ○ COCAINE
- ○ CRACK ou FREE BASE
- ○ ECSTASY ou MDMA
- ○ HEROINE
- ○ LSD
- ○ METHAMPHETAMINE
- ○ AUTRE

SI AUTRE SUBSTANCE, EXPLIQUER : ______________________

QUELLE A ETE LA FREQUENCE DE CETTE USAGE ?
- ○ AU MOINS 1 FOIS DEPUIS 3 MOIS
- ○ AU MOINS 1 FOIS PAR MOIS
- ○ AU MOINS 1 FOIS PAR SEMAINE
- ○ PLUSIEURS FOIS PAR SEMAINE
- ○ USAGE QUOTIDIEN

QUELS SONT LES EFFETS ATTENDUS ?
- ☐ PLAISIR / RECREATIF
- ☐ DEFONCE
- ☐ AUTOTHERAPEUTIQUE
- ☐ AUTRE

SI AUTRE EFFET ATTENDU, EXPLIQUER : ______________________

SI RECHERCHE D'EFFET THERAPEUTIQUE, QUEL(S) SYMPTOME(S) EST(SONT) SOULAGE(S) ?

☐ ANXIETE
☐ STRESS LIE AUX EXAMENS
☐ TROUBLE DU SOMMEIL
☐ DEPRESSION (HUMEUR TRISTE)
☐ DOULEUR PHYSIQUE
☐ AUTRE

SI AUTRE EFFET THERAPEUTIQUE RECHERCHE, EXPLIQUER : ________________________________

AVEZ-VOUS CONSOMME DANS LES 3 DERNIERS MOIS UNE AUTRE SUBSTANCE STUPEFIANTE ?

○ OUI ○ NON

SI OUI, LAQUELLE ?

○ AMPHETAMINE
○ CANNABIS (HERBE)
○ CANNABIS (RESINE)
○ CANNABINOIDES DE SYNTHESE (SPICE)
○ CATHINONES
○ CHAMPIGNONS HALLUCINOGENES
○ COCAINE
○ CRACK ou FREE BASE
○ ECSTASY ou MDMA
○ HEROINE
○ LSD
○ METHAMPHETAMINE
○ AUTRE

SI AUTRE SUBSTANCE, EXPLIQUER : ________________________________

QUELLE A ETE LA FREQUENCE DE CETTE USAGE ?

○ AU MOINS 1 FOIS DEPUIS 3 MOIS
○ AU MOINS 1 FOIS PAR MOIS
○ AU MOINS 1 FOIS PAR SEMAINE
○ PLUSIEURS FOIS PAR SEMAINE
○ USAGE QUOTIDIEN

QUELS SONT LES EFFETS ATTENDUS ?

☐ PLAISIR / RECREATIF
☐ DEFONCE
☐ AUTOTHERAPEUTIQUE
☐ AUTRE

SI AUTRE EFFET ATTENDU, EXPLIQUER : ________________________________

SI RECHERCHE D'EFFET THERAPEUTIQUE, QUEL(S) SYMPTOME(S) EST(SONT) SOULAGE(S) ?

☐ ANXIETE
☐ STRESS LIE AUX EXAMENS
☐ TROUBLE DU SOMMEIL
☐ DEPRESSION (HUMEUR TRISTE)
☐ DOULEUR PHYSIQUE
☐ AUTRE

SI AUTRE EFFET THERAPEUTIQUE RECHERCHE, EXPLIQUER : ________________________________

AVEZ-VOUS CONSOMME DANS LES 3 DERNIERS MOIS UNE AUTRE SUBSTANCE STUPEFIANTE ?

○ OUI ○ NON

SI OUI, LAQUELLE ?

○ AMPHETAMINE
○ CANNABIS (HERBE)
○ CANNABIS (RESINE)
○ CANNABINOIDES DE SYNTHESE (SPICE)
○ CATHINONES
○ CHAMPIGNONS HALLUCINOGENES
○ COCAINE
○ CRACK ou FREE BASE
○ ECSTASY ou MDMA
○ HEROINE
○ LSD
○ METHAMPHETAMINE
○ AUTRE

SI AUTRE SUBSTANCE, EXPLIQUER : ________________________________

QUELLE A ETE LA FREQUENCE DE CETTE USAGE ?
- ◯ AU MOINS 1 FOIS DEPUIS 3 MOIS
- ◯ AU MOINS 1 FOIS PAR MOIS
- ◯ AU MOINS 1 FOIS PAR SEMAINE
- ◯ PLUSIEURS FOIS PAR SEMAINE
- ◯ USAGE QUOTIDIEN

QUELS SONT LES EFFETS ATTENDUS ?
- ☐ PLAISIR / RECREATIF
- ☐ DEFONCE
- ☐ AUTOTHERAPEUTIQUE
- ☐ AUTRE

SI AUTRE EFFET ATTENDU, EXPLIQUER : ________________________________

SI RECHERCHE D'EFFET THERAPEUTIQUE, QUEL(S) SYMPTOME(S) EST(SONT) SOULAGE(S) ?
- ☐ ANXIETE
- ☐ STRESS LIE AUX EXAMENS
- ☐ TROUBLE DU SOMMEIL
- ☐ DEPRESSION (HUMEUR TRISTE)
- ☐ DOULEUR PHYSIQUE
- ☐ AUTRE

SI AUTRE EFFET THERAPEUTIQUE RECHERCHE, EXPLIQUER : ________________________________

AVEZ-VOUS CONSOMME DANS LES 3 DERNIERS MOIS UNE AUTRE SUBSTANCE STUPEFIANTE ? ◯ OUI ◯ NON

SI OUI, LAQUELLE ?
- ◯ AMPHETAMINE
- ◯ CANNABIS (HERBE)
- ◯ CANNABIS (RESINE)
- ◯ CANNABINOIDES DE SYNTHESE (SPICE)
- ◯ CATHINONES
- ◯ CHAMPIGNONS HALLUCINOGENES
- ◯ COCAINE
- ◯ CRACK ou FREE BASE
- ◯ ECSTASY ou MDMA
- ◯ HEROINE
- ◯ LSD
- ◯ METHAMPHETAMINE
- ◯ AUTRE

SI AUTRE SUBSTANCE, EXPLIQUER : ________________________________

QUELLE A ETE LA FREQUENCE DE CETTE USAGE ?
- ◯ AU MOINS 1 FOIS DEPUIS 3 MOIS
- ◯ AU MOINS 1 FOIS PAR MOIS
- ◯ AU MOINS 1 FOIS PAR SEMAINE
- ◯ PLUSIEURS FOIS PAR SEMAINE
- ◯ USAGE QUOTIDIEN

QUELS SONT LES EFFETS ATTENDUS ?
- ☐ PLAISIR / RECREATIF
- ☐ DEFONCE
- ☐ AUTOTHERAPEUTIQUE
- ☐ AUTRE

SI AUTRE EFFET ATTENDU, EXPLIQUER : ________________________________

SI RECHERCHE D'EFFET THERAPEUTIQUE, QUEL(S) SYMPTOME(S) EST(SONT) SOULAGE(S) ?
- ☐ ANXIETE
- ☐ STRESS LIE AUX EXAMENS
- ☐ TROUBLE DU SOMMEIL
- ☐ DEPRESSION (HUMEUR TRISTE)
- ☐ DOULEUR PHYSIQUE
- ☐ AUTRE

SI AUTRE EFFET THERAPEUTIQUE RECHERCHE, EXPLIQUER : ________________________________

AVEZ-VOUS CONSOMME DANS LES 3 DERNIERS MOIS UNE AUTRE SUBSTANCE STUPEFIANTE ? ◯ OUI ◯ NON

SI OUI, LAQUELLE ?
- ◯ AMPHETAMINE
- ◯ CANNABIS (HERBE)
- ◯ CANNABIS (RESINE)
- ◯ CANNABINOIDES DE SYNTHESE (SPICE)
- ◯ CATHINONES
- ◯ CHAMPIGNONS HALLUCINOGENES
- ◯ COCAINE
- ◯ CRACK ou FREE BASE
- ◯ ECSTASY ou MDMA
- ◯ HEROINE
- ◯ LSD
- ◯ METHAMPHETAMINE
- ◯ AUTRE

SI AUTRE SUBSTANCE, EXPLIQUER : __________________________________

QUELLE A ETE LA FREQUENCE DE CETTE USAGE ?
- ◯ AU MOINS 1 FOIS DEPUIS 3 MOIS
- ◯ AU MOINS 1 FOIS PAR MOIS
- ◯ AU MOINS 1 FOIS PAR SEMAINE
- ◯ PLUSIEURS FOIS PAR SEMAINE
- ◯ USAGE QUOTIDIEN

QUELS SONT LES EFFETS ATTENDUS ?
- ☐ PLAISIR / RECREATIF
- ☐ DEFONCE
- ☐ AUTOTHERAPEUTIQUE
- ☐ AUTRE

SI AUTRE EFFET ATTENDU, EXPLIQUER : __________________________________

SI RECHERCHE D'EFFET THERAPEUTIQUE, QUEL(S) SYMPTOME(S) EST(SONT) SOULAGE(S) ?
- ☐ ANXIETE
- ☐ STRESS LIE AUX EXAMENS
- ☐ TROUBLE DU SOMMEIL
- ☐ DEPRESSION (HUMEUR TRISTE)
- ☐ DOULEUR PHYSIQUE
- ☐ AUTRE

SI AUTRE EFFET THERAPEUTIQUE RECHERCHE, EXPLIQUER : __________________________________

AVEZ-VOUS CONSOMME DANS LES 3 DERNIERS MOIS UNE AUTRE SUBSTANCE STUPEFIANTE ? ◯ OUI ◯ NON

SI OUI, LAQUELLE ?
- ◯ AMPHETAMINE
- ◯ CANNABIS (HERBE)
- ◯ CANNABIS (RESINE)
- ◯ CANNABINOIDES DE SYNTHESE (SPICE)
- ◯ CATHINONES
- ◯ CHAMPIGNONS HALLUCINOGENES
- ◯ COCAINE
- ◯ CRACK ou FREE BASE
- ◯ ECSTASY ou MDMA
- ◯ HEROINE
- ◯ LSD
- ◯ METHAMPHETAMINE
- ◯ AUTRE

SI AUTRE SUBSTANCE, EXPLIQUER : __________________________________

QUELLE A ETE LA FREQUENCE DE CETTE USAGE ?
- ◯ AU MOINS 1 FOIS DEPUIS 3 MOIS
- ◯ AU MOINS 1 FOIS PAR MOIS
- ◯ AU MOINS 1 FOIS PAR SEMAINE
- ◯ PLUSIEURS FOIS PAR SEMAINE
- ◯ USAGE QUOTIDIEN

QUELS SONT LES EFFETS ATTENDUS ?
- ☐ PLAISIR / RECREATIF
- ☐ DEFONCE
- ☐ AUTOTHERAPEUTIQUE
- ☐ AUTRE

SI AUTRE EFFET ATTENDU, EXPLIQUER : ______________________________

SI RECHERCHE D'EFFET THERAPEUTIQUE, QUEL(S) SYMPTOME(S) EST(SONT) SOULAGE(S) ?

- ☐ ANXIETE
- ☐ STRESS LIE AUX EXAMENS
- ☐ TROUBLE DU SOMMEIL
- ☐ DEPRESSION (HUMEUR TRISTE)
- ☐ DOULEUR PHYSIQUE
- ☐ AUTRE

SI AUTRE EFFET THERAPEUTIQUE RECHERCHE, EXPLIQUER : ______________________________

3. STRESS ET FATIGUE

MERCI DE REPONDRE AUX QUESTIONS SUIVANTES

EVALUER VOTRE NIVEAU DE STRESS DES 7 DERNIERS JOURS :

AUCUN STRESS — STRESS MAXIMAL IMAGINABLE

(Place a mark on the scale above)

EVALUER VOTRE NIVEAU DE FATIGUE DES 7 DERNIERS JOURS :

AUCUNE FATIGUE — FATIGUE MAXIMALE IMAGINABLE

(Place a mark on the scale above)

4. ANXIETE ET DEPRESSION

MERCI DE REPONDRE AUX QUESTIONS SUIVANTES

JE ME SENS TENDU OU ENERVE

- ◯ LA PLUPART DU TEMPS
- ◯ SOUVENT
- ◯ DE TEMPS EN TEMPS
- ◯ JAMAIS

J'AI TOUJOURS AUTANT DE PLAISIR A FAIRE LES CHOSES QUI ME PLAISENT HABITUELLEMENT

- ◯ OUI, TOUT AUTANT
- ◯ PAS AUTANT
- ◯ UN PEU SEULEMENT
- ◯ PRESQUE PLUS DU TOUT

J'AI UNE SENSATION DE PEUR COMME SI QUELQUE CHOSE D'HORRIBLE ALLAIT M'ARRIVER

- ◯ OUI, TRES NETTEMENT
- ◯ OUI, MAIS CE N'EST PAS TROP GRAVE
- ◯ UN PEU, MAIS CELA NE M'INQUIETE PAS
- ◯ PAS DU TOUT

JE SAIS RIRE ET VOIR LE COTE AMUSANT DES CHOSES

- ◯ TOUJOURS AUTANT
- ◯ PLUTOT MOINS
- ◯ NETTEMENT MOINS
- ◯ PLUS DU TOUT

JE ME FAIS DU SOUCI

- ◯ TRES SOUVENT
- ◯ ASSEZ SOUVENT
- ◯ OCCASIONNELLEMENT
- ◯ TRES OCCASIONNELLEMENT

JE ME SENS GAI ET DE BONNE HUMEUR

- ◯ JAMAIS
- ◯ RAREMENT
- ◯ ASSEZ SOUVENT
- ◯ LA PLUPART DU TEMPS

JE PEUX RESTER TRANQUILLEMENT ASSIS AU REPOS ET ME SENTIR DETENDU

◯ JAMAIS
◯ RAREMENT
◯ OUI, EN GENERAL
◯ OUI, TOUJOURS

J'AI L'IMPRESSION DE FONCTIONNER AU RALENTI

◯ PRATIQUEMENT TOUT LE TEMPS
◯ TRES SOUVENT
◯ QUELQUEFOIS
◯ JAMAIS

J'EPROUVE DES SENSATIONS DE PEUR ET J'AI L'ESTOMAC NOUE

◯ TRES SOUVENT
◯ ASSEZ SOUVENT
◯ PARFOIS
◯ JAMAIS

JE NE M'INTERESSE PLUS A MON APPARENCE

◯ TOTALEMENT
◯ JE N'Y FAIS PLUS ATTENTION
◯ JE N'Y FAIS PLUS ASSEZ ATTENTION
◯ J'Y FAIS ATTENTION COMME D'HABITUDE

JE NE TIENS PAS EN PLACE

◯ OUI, C'EST TOUT A FAIT LE CAS
◯ UN PEU
◯ PAS TELLEMENT
◯ PAS DU TOUT

JE ME REJOUIS A L'AVANCE DE FAIRE CERTAINES CHOSES

◯ COMME D'HABITUDE
◯ PLUTOT MOINS QU'AVANT
◯ BEAUCOUP MOINS QU'AVANT
◯ PAS DU TOUT

J'EPROUVE DES SENSATIONS SOUDAINES DE PANIQUE

◯ TRES SOUVENT
◯ ASSEZ SOUVENT
◯ RAREMENT
◯ JAMAIS

JE PEUX PRENDRE PLAISIR A LIRE UN BON LIVRE OU A ECOUTER UNE BONNE EMISSION DE RADIO OU DE TELEVISION

◯ SOUVENT
◯ PARFOIS
◯ RAREMENT
◯ PRATIQUEMENT JAMAIS

5. CURSUS UNIVERSITAIRES

MERCI DE REPONDRE AUX QUESTIONS SUIVANTES

ANNEE D'ETUDE :

◯ PACES
◯ DFGSP2
◯ DFGSP3
◯ DFASP1
◯ DFASP2 OFFICINE
◯ DFASP2 INDUSTRIE
◯ DFASP2 RECHERCHE
◯ DFASP2 INTERNAT
◯ 6eme ANNEE OFFICINE
◯ 6eme ANNEE INDUSTRIE
◯ 6eme ANNEE RECHERCHE

REGION DE L'UNIVERSITE D'APPARTENANCE :

- ◯ ALSACE
- ◯ AQUITAINE
- ◯ AUVERGNE
- ◯ BASSE-NORMANDIE
- ◯ BOURGOGNE
- ◯ BRETAGNE
- ◯ CENTRE-VAL DE LOIRE
- ◯ CHAMPAGNE-ARDENNE
- ◯ CORSE
- ◯ FRANCHE-COMTE
- ◯ HAUTE-NORMANDIE
- ◯ ILE-DE-FRANCE
- ◯ LANGUEDOC-ROUSSILLON
- ◯ LIMOUSIN
- ◯ LORRAINE
- ◯ MIDI-PYRENEES
- ◯ NORD-PAS-DE-CALAIS
- ◯ PAYS DE LA LOIRE
- ◯ PICARDIE
- ◯ POITOU-CHARENTES
- ◯ PROVENCE-ALPES-COTE D'AZUR
- ◯ RHONE-ALPES

QUELLE A ETE VOTRE MOYENNE ANNUELLE L'ANNEE DERNIERE ?

- ◯ MOINS DE 10
- ◯ ENTRE 10 ET 12
- ◯ ENTRE 12 ET 14
- ◯ ENTRE 14 ET 16
- ◯ PLUS DE 16

(NOTE SUR 20)

EVALUER LE NIVEAU DE DIFFICULTE DE VOS ETUDES :

ETUDE FACILE — ETUDE TRES DIFFICILE

(Place a mark on the scale above)

EVALUER VOTRE NIVEAU DE PRESENTEISME EN COURS :

PRESENCE A AUCUN COURS — PRESENCE A TOUS LES COURS

(Place a mark on the scale above)

AVEZ-VOUS DEJA REDOUBLE DURANT VOS ETUDES DE PHARMACIE ?

◯ OUI ◯ NON

QUELLE(S) ANNEE(S) AVEZ-VOUS REDOUBLEE(S) ?

- ◯ PACES
- ◯ DFGSP2
- ◯ DFGSP3
- ◯ DFASP1
- ◯ DFASP2
- ◯ 6eme ANNEE

QUAND ONT EU LIEU LES DERNIERS EXAMENS ?

- ◯ MOINS DE 1 MOIS
- ◯ PLUS DE 1 MOIS

QUAND AURONT LIEU LES PROCHAINS EXAMENS ?

- ◯ L'ANNEE EST TERMINEE
- ◯ DANS MOINS DE 1 MOIS
- ◯ DANS PLUS DE 1 MOIS

6. CARACTERISTIQUES PERSONNELLES

MERCI DE REPONDRE AUX QUESTIONS SUIVANTES

GENRE

○ HOMME ○ FEMME

AGE ______________________ (ans)

CONSOMMEZ-VOUS DES CIGARETTES QUOTIDIENNEMENT ? ○ OUI ○ NON

CONSOMMEZ-VOUS DES CIGARETTES ELECTRONIQUES QUOTIDIENNEMENT ? ○ OUI ○ NON

COMPOSITION DE LA CIGARETTE ELECTRONIQUE ○ AVEC NICOTINE ○ SANS NICTOINE

CONSOMMEZ-VOUS, MEME OCCASIONNELLEMENT, DES BOISSONS ALCOOLISEES ? ○ OUI ○ NON

CONSOMMEZ-VOUS PLUS DE 3 VERRES D'ALCOOL PAR JOUR ET/OU PLUS DE 21 VERRES D'ALCOOL PAR SEMAINE ? ○ OUI ○ NON

CONSOMMEZ-VOUS PLUS DE 2 VERRES D'ALCOOL PAR JOUR ET/OU PLUS DE 14 VERRES D'ALCOOL PAR SEMAINE ? ○ OUI ○ NON

COMMENTAIRE LIBRE ______________________
